# Prostaglandin EP4 Selective Agonist AKDS001 Enhances New Bone Formation by Minimodeling in a Rat Heterotopic Xenograft Model of Human Bone

**DOI:** 10.3389/fbioe.2022.845716

**Published:** 2022-03-17

**Authors:** Yuichiro Ukon, Masahiro Nishida, Natsumi Yamamori, Kazuhiro Takeyama, Kazuhito Sakamoto, Shota Takenaka, Takahiro Makino, Takahito Fujimori, Yusuke Sakai, Yuya Kanie, Joe Kodama, Zeynep Bal, Daisuke Tateiwa, Shinichi Nakagawa, Hiromasa Hirai, Seiji Okada, Takashi Kaito

**Affiliations:** ^1^ Department of Orthopaedic Surgery, Osaka University Graduate School of Medicine, Osaka, Japan; ^2^ Laboratory for Pharmacology, Pharmaceuticals Research Center, Asahi Kasei Pharma Corporation, Shizuoka, Japan; ^3^ Department of Orthopaedic Surgery, Hayaishi Hospital, Osaka, Japan; ^4^ Department of Orthopaedic Surgery, Suita Municipal Hospital, Osaka, Japan; ^5^ Department of Orthopedics, University of Maryland School of Medicine, Baltimore, MD, United States; ^6^ Department of Signal Transduction, Research Institute for Microbial Diseases, Osaka University, Osaka, Japan

**Keywords:** xenograft, EP4 agonist, prostaglandin, bone grafting, autograft, autogenous bone, spinal fusion

## Abstract

To enhance bone regeneration, the use of bone morphogenetic protein (BMP)-2 is an attractive option. Unfortunately, the dose-dependent side effects prevent its widespread use. Therefore, a novel osteogenic agent using a different mechanism of action than BMP-2 is highly desirable. Previous reports demonstrated that prostaglandin E2 receptor 4 (EP4) agonists have potent osteogenic effects on non-human cells and are one of the potential alternatives for BMP-2. Here, we investigated the effects of an EP4 agonist (AKDS001) on human cells with a rat heterotopic xenograft model of human bone. Bone formation in the xenograft model was significantly enhanced by AKDS001 treatment. Histomorphometric analysis showed that the mode of bone formation by AKDS001 was minimodeling rather than remodeling. In cultured human mesenchymal stem cells, AKDS001 enhanced osteogenic differentiation and mineralization *via* the cAMP/PKA pathway. In cultured human preosteoclasts, AKDS001 suppressed bone resorption by inhibiting differentiation into mature osteoclasts. Thus, we conclude that AKDS001 can enhance bone formation in grafted autogenous bone by minimodeling while maintaining the volume of grafted bone. The combined use of an EP4 agonist and autogenous bone grafting may be a novel treatment option to enhance bone regeneration. However, we should be careful in interpreting the results because male xenografts were implanted in male rats in the present study. It remains to be seen whether females can benefit from the positive effects of AKDS001 MS by using female xenografts implanted in female rats in clinically relevant animal models.

## 1 Introduction

The gold standard grafting material for large bone defects or spinal fusion surgeries is autogenous bone (Bauer and Muschler, 2000; García-Gareta et al., 2015). However, autogenous bone is associated with several disadvantages, including the amount of bone, donor site morbidity, and ageing-related deterioration of bone quality (García-Gareta et al., 2015). To overcome these disadvantages, a multitude of biomaterials and osteogenic agents to enhance bone regeneration were developed (Dimitriou et al., 2011; García-Gareta et al., 2015).

Among osteogenic agents, the use of bone morphogenetic protein (BMP)-2 is most prevalent, especially in spinal fusion surgeries (Makino et al., 2018; Ukon et al., 2019). However, the potential side effects related to BMP-2 use, such as soft-tissue swelling, osteoclastic bone resorption, and unintended ectopic bone formation, limit its widespread use (James et al., 2016). Therefore, there is a strong need for the development of a novel osteogenic agent that acts through a different mechanism of action.

Prostaglandin E2 (PGE2), which is synthesized from arachidonic acid by cyclooxygenases, is also known to have potent osteogenic effects (Raisz et al., 1993; Li et al., 2007). PGE2-sensitive receptors, which are termed EP, have been subdivided into EP1, EP2, EP3, and EP4. Among these, EP4 receptor is known to be associated with the effects of PGE2 on bone metabolism (Raisz et al., 1993; Li et al., 2007). Over the past two decades, several EP4 receptor-selective agonists have been developed and there is accumulating evidence that EP4 agonists have therapeutic potential for the treatment of osteoporosis and for bone healing in preclinical research using animal models or non-human cells (Toyoda et al., 2005; Ke et al., 2006; Iwaniec et al., 2007; Nakagawa et al., 2007; Liu et al., 2015). The efficacy of EP4 agonists in human bone tissue, however, has not yet been proven. Therefore, in this study, we investigated the effects of an EP4 agonist (AKDS001) on human cells and human bone tissue with a newly established rat heterotopic xenograft model and explored the therapeutic potential of this EP4 agonist as an osteogenic enhancer in combination with autogenous bone grafting.

## 2 Materials and Methods

### 2.1 Ethics Statement

All experiments were approved by the institutional review board (No. 17027-2) and the institutional animal committee of our institution. Human vertebral laminas were obtained from five human donors who underwent lumbar decompression surgery for degenerative spinal disease (Table 1). All donor patients provided informed consent for the experiments.

**TABLE 1:** Detailed information about the xenotransplantation of human vertebral bone.

Donors	Recipient Rats	Number of implanted samples	Concentration of AKDS001	Analyses
67-year-old man	No. 1	12	0 (Blank) and 1.0 mg/ml (*n* = 6 for each concentration)	μCT + histology
68-year-old man	No. 2			μCT
79-year-old man	No. 3			μCT
70-year-old man	No. 4			Histomorphometry
83-year-old man	No. 5		0 (Blank), 0.1, 0.3 and 1.0 mg/ml (*n* = 6 for each concentration)	Histomorphometry
	No. 6			Histomorphometry

### 2.2 Chemicals

The selective EP4 agonist, AKDS001 (Asahi Kasei Pharma, Tokyo, Japan), was incorporated into biocompatible and biodegradable polylactic-co-glycolic acid (PLGA) microspheres (MS) for sustained drug release (Hua et al., 2021). The ratio of polylactic acid to polyglycolic acid was 1:1. PLGA MS with AKDS001 (AKDS001 MS) and without AKDS001 (Blank MS) were used for the following animal experiments. Approximately 70% of AKDS001 in PLGA MS was released sustainedly over the study period (4 weeks) (Ukon et al., 2021).

### 2.3 Animals

Six male 6-week-old F344/Njcl-rnu/rnu rats (CLEA Japan, Tokyo, Japan), which lack T-cell function, were used. The age of the rats was decided as per previous reports (Yoshikawa et al., 1996; Surowiec et al., 2020). Rats were anesthetized with an intraperitoneal injection of 0.3 mg/kg medetomidine (Nippon Zenyaku Kogyo, Fukushima, Tokyo, Japan), 4.0 mg/kg midazolam (Astellas Pharma, Tokyo, Japan), and 5.0 mg/kg butorphanol (Meiji Seika Pharma, Tokyo, Japan).

### 2.4 *In vivo* Experiments

#### 2.4.1 Preparation of *in Vitro*–Cultured Bone Grafts From human Vertebral Lamina Bone

**FIGURE 1:**
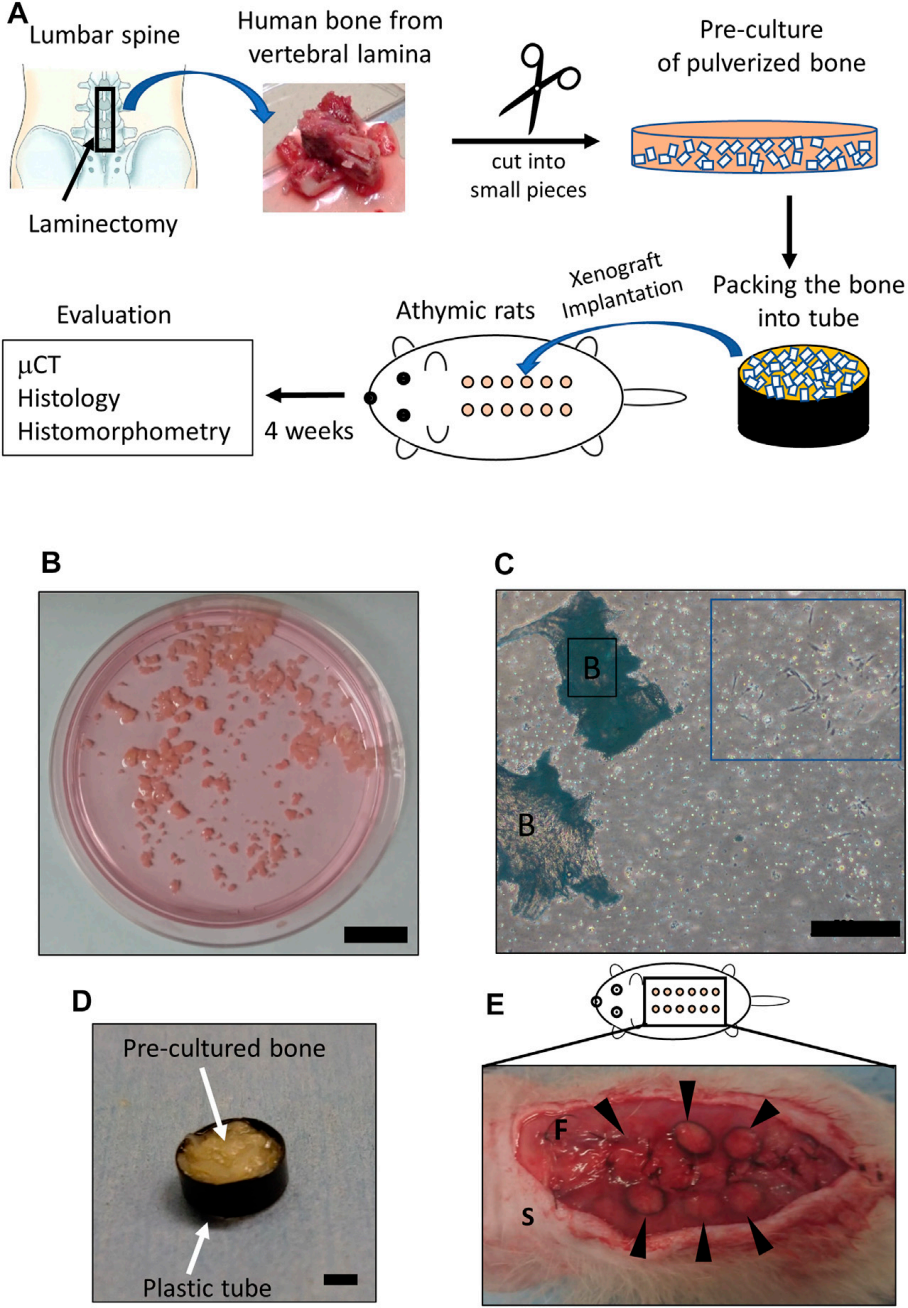
Details of the xenograft model. **(A)** Scheme showing the protocol of the xenograft model. The square indicates the area where vertebral bone was obtained (upper left). Bone of vertebral lamina (upper center) was pulverized into small pieces (≤1 mm) (upper right) and pre-cultured for 2 weeks. Twelve xenograft samples (circles colored in orange) were implanted underneath the dorsal fascia (lower center). Evaluations were performed 4 weeks after implantation. **(B)** Pre-culture of pulverized bone in medium. Scale bar, 2 mm. **(C)** Human cells observed 2 weeks after pre-culture (blue square). Scale bars, 500 μm. B, bone. **(D)** Pre-cultured bone filling a plastic tube. The plastic tube and the packed in pre-cultured bone are indicated by arrows. Scale bar, 2 mm. **(E)** Subfascial transplantation of xenografted bone. Arrowheads indicate xenografted samples. S, skin; F, fascia.

The overall protocol of the xenograft model is shown in Figure 1A. Human bones of vertebral laminas were obtained by laminectomy during lumbar decompression surgery (Figure 1A). Bone from older adult patients was used because current BMP-2 use for bone regeneration is targeted at older adults who need spinal fusion. Human cancellous bones of vertebra laminas were preprocessed according to the previously published method (Dillon et al., 2012). In brief, human cancellous bone was isolated by removing the cortical bone and was then pulverized into small pieces (≤1 mm) with scissors. The extracted cancellous bone was rinsed with phosphate-buffered saline (PBS) and was cultured at a density of 0.3 g of tissue/100-mm-diameter Petri dish with αMEM containing 10% fetal bovine serum (FBS) and 1% penicillin/streptomycin, 50 μg/μL ascorbic acid, 10 mM β-glycerol phosphate, and 100 nM dexamethasone as per previous reports (Figure 1B) (Yoshikawa et al., 1996; Yoshikawa and Myoui, 2005). After culturing for 7 days, the medium was changed every 3 days. Human bone cells that migrated from the bone chips were observed at 7–10 days (Figure 1C). After a total of 14 days of explant culture, the bone chips in the media were collected and packed into plastic tubes (diameter: 6 mm, height: 1 mm) for the xenotransplantation (Figure 1D).

#### 2.4.2 Xenotransplantation of Human Vertebral Lamina Bone Into Athymic Nude Rats

Six male 6-week-old athymic nude congenic Fischer (F344/Njcl-rnu/rnu) rats were used for the following experiments: After a posterior midline skin incision under anesthesia, the xenograft samples prepared in the manner described above were implanted underneath the right (*n* = 6) and left (*n* = 6) fascia of the dorsal muscle (a total of 12 samples per rat) (Figure 1E). The experiments used four rats in which 48 xenograft samples obtained from four patients were implanted and two rats in which 24 xenograft samples obtained from one patient were implanted (Table 1). Blank MS or AKDS001 MS was mixed with the *in vitro–*cultured bone grafts (*n* = 6 for each). Fascia and skin were closed with 4-0 nylon. Antibiotics (penicillin G, 10,000 U/kg) were subcutaneously injected for 3 days after surgery. All rats were euthanized 4 weeks after the surgery because the sustained release of AKDS001 MS was confirmed up to 4 weeks. The excised xenograft samples were used for microcomputed tomography (μCT), histology, and histomorphometry analyses.

#### 2.4.3 μCT Analysis

Two high-resolution μCT machines (Skyscan 1272, Bruker, Billerica, MA, United States; and R_mCT, Rigaku, Tokyo, Japan) were used. Pre- and post-operative samples were scanned by R_mCT and Skyscan1272, respectively. R_mCT and Skyscan1272 were used at a resolution of 20 and 5 μm per voxel, respectively. Bone microstructural analysis of the data from R_mCT was performed with TRI/3D-BON64 software (RATOC System Engineering, Tokyo, Japan). The data from Skyscan1272 was analyzed by the CTAn (Bruker). The newly formed bone (woven bone) area was extracted from Skyscan1272 data by the analysis processing used in the previous report (Kodama et al., 2021). Briefly, processing is based on Hounsfield unit (HU) value. The accuracy of the extracted new bone area by the analysis processing was validated by matching the woven bone area on the corresponding histological sections (Supplementary Figures S1A–E).

#### 2.4.4 Histological and Immunohistochemical Analysis

The excised samples were fixed in formalin, EDTA-decalcified, embedded in paraffin wax, and cut at 3 μm thickness. Hematoxylin and eosin (HE) staining, EP4 (Novus Biologicals, NLS3890), CD31 (Abcam, ab182981), human-vimentin (Abcam, ab16700), human-osteocalcin (Takara Bio, M184), and rat-osteocalcin (Takara Bio, M186) immunostaining were performed with the standard protocols. Immunostaining for human-vimentin, human-osteocalcin, and rat-osteocalcin was assessed with human vertebral lamina and rat femur as controls (Supplementary Figure S2).

#### 2.4.5 Histomorphometric Analysis

Double labeling was performed by subcutaneous injection of tetracycline (20 mg/kg) and calcein (10 mg/kg) at 5 and 2 days before euthanasia, respectively. Extracted samples were fixed by 70% ethanol and were treated with Villanueva bone stain, and then embedded in methacrylate (Wako Pure Chemical Industries, Kanagawa, Japan). Then, the following histomorphometric parameters were quantified: newly formed bone volume (NBV), number of osteoblasts (N.Ob), osteoblast surface (Ob.S), number of osteoblasts per bone surface (N.Ob/BS), osteoblast surface per bone surface (Ob.S/BS), mineral apposition rate (MAR), bone formation rate per bone surface (BFR/BS), number of osteoclasts per bone surface (N.Oc/BS), and osteoclast surface per bone surface (Oc.S/BS).

### 2.5 *In vitro* Experiments

#### 2.5.1 Binding Assay

Affinities and selectivity of AKDS001 for human EP were evaluated using human embryonic kidney (HEK)-293 cells stably expressing human EP, as described in a previous report (Suzawa et al., 2000). In brief, EP cDNAs subcloned into a pCEP4 vector (Invitrogen, San Diego, CA, United States) were transfected into HEK cells. HEK cells expressing the cDNA together with the hygromycin resistance gene were selected and expanded into clonal cell lines. Membranes were prepared by centrifugation following lysis of the cells by nitrogen cavitation. Assays were performed in a final incubation volume of 0.2 ml in 10 mM MES/KOH containing 1 mM EDTA, 10 mM MgCl_2_, and [3H] PGE2 (181 Ci/mmol) (1.5 nM for EP1, 3 nM for EP2, and 0.5 nM for EP3 and EP4). The reaction was initiated by the addition of membranes expressing human EP (30 μg for EP1, 20 μg for EP2, 2 μg for EP3, and 10 μg for EP4) and AKDS001. Non-specific binding was determined in the presence of non-radioactive PGE2 (10 μM for EP1, EP2, and EP4, and 1 μM for EP3). Incubations were conducted for 120 min at room temperature and the assays were terminated by filtration through a 96-well Unifilter GF/C. The filters were washed and dried, and the residual radioactivity was determined by liquid scintillation counting.

#### 2.5.2 Adenosine 3′, 5′-Cyclic Monophosphate (cAMP) Assay Using Stably Expressing Cells of Human EP

The increasing effect of AKDS001 or PGE2 on cAMP production was evaluated using Chinese hamster ovary (CHO) cells stably expressing human EP2, EP4, or IP receptors (Wilson et al., 2004). In brief, EP2, EP4, or IP receptor cDNAs were subcloned into the pcDNA3 vector (Invitrogen, Waltham, MA, United States) and transfected into CHO cells. CHO cells expressing the cDNA together with the neomycin resistance gene were selected and expanded into clonal cell lines. Cells were incubated with AKDS001 or PGE2. The amount of cAMP was assessed by a homogeneous time-resolved fluorescence assay.

#### 2.5.3 cAMP Assay Using CHO-K1 Cells Transiently Expressing Human and Rat EP4

CHO-K1 cells were transfected with a human or rat EP4 expression vector subcloned into the pcDNA3 vector (Invitrogen) using Lipofectamine 3000 (Invitrogen) and incubated for 16–24 h. Then, 4,000 cells/well of these cells were treated with AKDS001 or PGE2 at concentrations of 1 × 10^–15^ to 1 × 10^–5^ mol/L for 30 min in a 384-well plate. The amount of cAMP was assessed using a LANCE^TM^ Ultra cAMP kit (PerkinElmer, Waltham, MA, United States) according to the manufacturer’s protocols.

#### 2.5.4 Osteoblastic Differentiation of Human Bone Marrow-Derived Mesenchymal Stem Cells

The overall protocol of the osteoblastic differentiation assay is shown in Figure 6A. Human bone marrow–derived mesenchymal stem cells (hMSCs) from three donors were purchased from Lonza (Cologne, Germany) and cultured independently. hMSCs were seeded at 10,000 cells/well on a 48-well Biocoat Collagen I plate and cultured in a growth medium (MSC Growth Medium 2 and 1% penicillin-streptomycin) for 3 days. Confluent hMSCs were cultured in osteogenic differentiation medium (OM) (DMEM containing 10% FBS, 1% penicillin-streptomycin, 50 μg/mL L-ascorbic acid, 10 mM β-glycerol phosphate, 100 nM dexamethasone, and 100 ng/ml BMP-2) for 3 days, and then AKDS001 (100 pM–100 nM) or 0.1% dimethyl sulfoxide solution (DMSO) was added to the OM. The medium was changed every 3–4 days.

#### 2.5.5 Assay for Alkaline Phosphatase Activity

Alkaline phosphatase (ALP) activity was determined after 7 days of culture with AKDS001 or DMSO. The cultured cells were washed once with PBS, solubilized with 0.05% Triton X/PBS solution, frozen, and then thawed. The cells were subsequently sonicated and centrifuged, and supernatants were measured for ALP activity using LaboAssay^TM^ ALP (FUJIFILM Wako Pure Chemical Corp., Osaka, Japan) according to the manufacturer’s protocol. One unit of activity was defined as 1 nmol of p-nitrophenol in 1 min in a reaction carried out at pH9.8 and 37°C. The activity was normalized by protein content assayed with a Pierce^TM^ BCA Protein Assay Kit (Thermo Fisher, Waltham, MA, United States). This assay was performed in triplicate. ALP activity in the treatment groups (AKDS001 0.1–100 nM) was calculated as the relative value to that of the control group (AKDS001 0 nM) for each donor, and the effects of AKDS treatments on ALP activity were investigated.

#### 2.5.6 Quantification of Mineralization

Mineralization by hMSC-derived osteoblasts was assessed after 14 days of culture with AKDS001 or DMSO using a Mineralization Evaluation Set (PG Research, Tokyo, Japan). The cultured cells were washed twice with PBS and fixed with 4% PFA for 20 min. After washing twice with PBS, matrix mineralization was stained using an alizarin red solution and quantified by measuring the absorbance at 415 nm according to the manufacturer’s protocol. This experiment was performed in triplicate. Absorbance of mineralization in the treatment groups (AKDS001 0.1–100 nM) was calculated as the relative value to that of the control group (AKDS001 0 nM) for each donor, and the effects of AKDS treatments on mineralization were investigated.

#### 2.5.7 Inhibitory Analysis of cAMP Signaling Pathway

H89 (Cayman Chemical, Ann Arbor, MI, United States), the PKA inhibitor, and wortmannin (FUJIFILM Wako Pure Chemical Corp.), the PI3K inhibitor, were used. hMSCs were seeded at 10,000 cells/well on a 48-well Biocoat Collagen I plate and cultured in a growth medium for 3 days. Confluent hMSCs were cultured in the OM for 3 days and then AKDS001 (100 nM) and H89 (0, 5, 10, and 20 μM) or wortmannin (0, 20, 100, and 500 nM) were added to the medium. The medium was changed every 3–4 days. ALP activity was determined after 7 days of culture with AKDS001 or DMSO.

#### 2.5.8 Membrane-Permeable cAMP Analog Assay

Dibutyryl-cAMP (Sigma-Aldrich, Saint Louis, MO, United States) was used as the membrane-permeable cAMP analog. hMSCs were seeded at 10,000 cells/well on a 48-well Biocoat Collagen I plate and cultured in growth medium for 3 days. Confluent hMSCs were cultured in the OM medium for 3 days and then dibutyryl-cAMP (0, 0.5, 1, and 2 mM) was added to the medium. The medium was changed every 3–4 days. ALP activity was determined after 7 days of culture with cAMP analog.

#### 2.5.9 cAMP Reactivity Assay of hMSCs to AKDS001

hMSCs were seeded at 10,000 cells/well on 6-well plates and cultured for 16–24 h. Then, 500 cells/well of these cells were treated with AKDS001 at concentrations of 1 × 10^–11^ to 1 × 10^–6^ mol/L for 30 min in a 384-well plate and the amount of cAMP was assessed using a LANCE^TM^ Ultra cAMP kit (PerkinElmer) according to the manufacturer’s protocols.

#### 2.5.10 Osteoclastic Differentiation Assay

Osteoclast precursors were isolated from human peripheral blood obtained from 38- and 57-year-old healthy male volunteers. Mononuclear cells were obtained by Ficoll-Hypaque centrifugation and CD14^+^ monocytes were isolated as human osteoclast precursors using MACS (Miltenyi Biotec, Bergisch, Germany). The cells were cultured in an αMEM medium containing 10% FBS, 25 ng/mL M-CSF, and 100 ng/ml receptor activator of NF-κB ligand (RANKL medium) on 96-well plates, and then AKDS001 (100 pM–1 μM) or 0.1% DMSO was added to the RANKL medium. After 5 days culture, multinucleated cells of three or more nuclei stained with an Acid phosphatase Leukocyte (TRAP) Kit (Sigma-Aldrich) were counted under a light microscope.

#### 2.5.11 Osteoclastic Activity Assay

The CD14-positive monocytes were cultured in the RANKL medium on 6-well plates for 5 days for the osteoclastogenesis. Multinucleated cells were detached and seeded in RANKL medium containing AKDS001 (100 pM–1 μM) or 0.1% DMSO on a 96-well plastic plate and a 96-well plate with a mineral matrix (Corning osteoassay surface 96-well multiple well plate, Corning, NY, United States), respectively. After 48 h in the plastic plate, cells were stained for TRAP, and the number of mature osteoclasts (TRAP-positive cells with 3 or more nuclei) was counted by light microscopy in each well. In the plate coated with the mineral matrix, cells were lysed, and the extent of resorbed areas was quantified using software dedicated to image analysis. Results are expressed in percent of resorption per 100 osteoclasts. Microscopic image acquisition was performed using a Nikon Eclipse 80i (Tokyo, Japan). Reconstruction of images and determination of the extent of resorbed areas were performed using Nikon NIS-D software. The extent of the white areas corresponding to the area of mineral matrix resorbed by mature osteoclasts was determined manually.

### 2.6 Statistical Analysis

Statistical analysis was performed using GraphPad Prism version 8.4.1 for Windows (GraphPad Software, San Diego, California, United States). Two groups were compared by an unpaired Student’s *t*- or Mann–Whitney *U* test; and more than three groups were compared by one-way analysis of variance and Dunnett’s multiple comparison. The values are presented as the mean ± SD. A *p*-value < 0.05 was considered statistically significant.

## 3 Results

### 3.1 Human Osteoblasts Express EP4 Receptor

**FIGURE 2:**
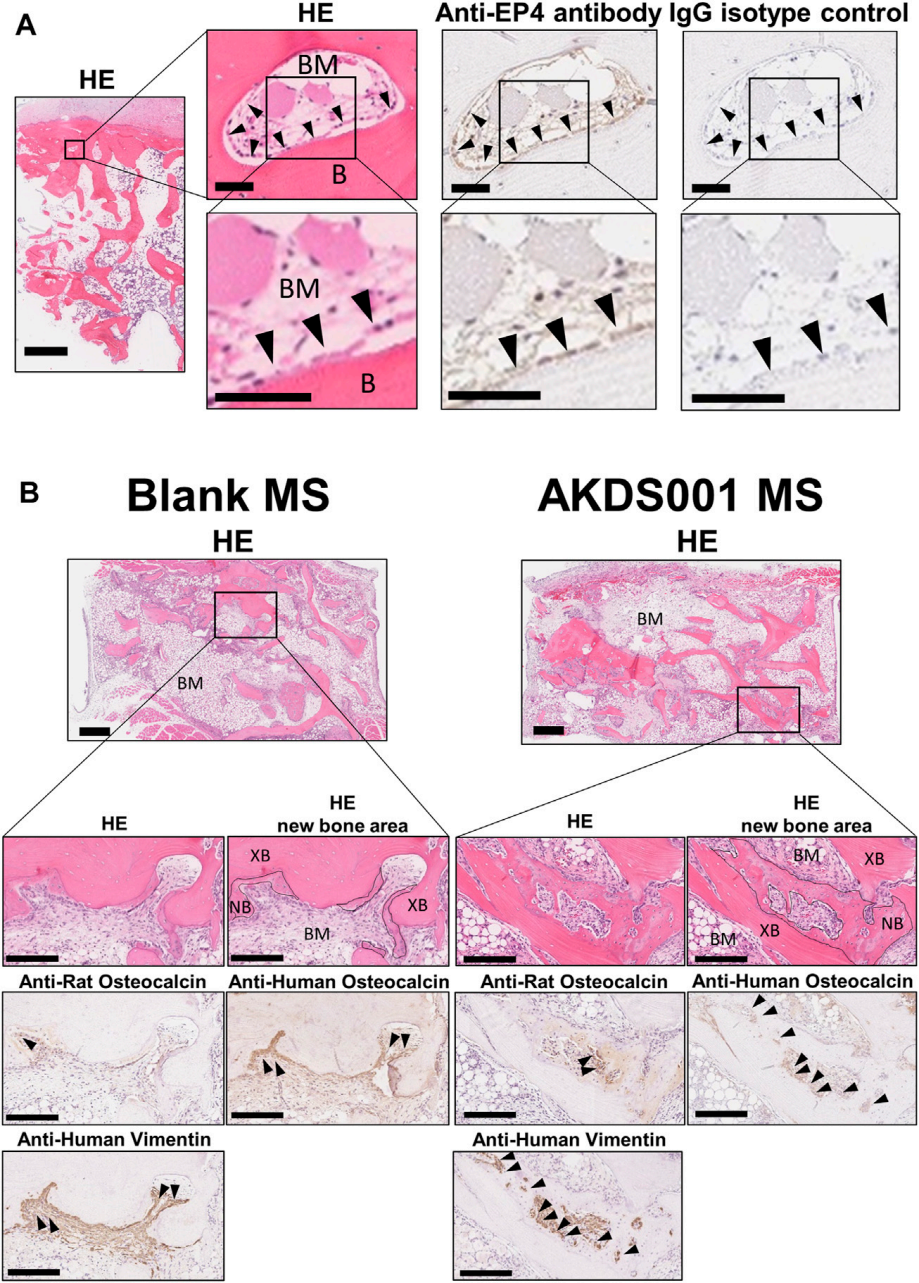
Histological images of human bone and extracted samples. **(A)** Representative images of hematoxylin and eosin (HE) staining, and anti-EP4 and IgG control immunostaining of human vertebral bone. Arrowheads indicate representative anti-EP4 immunostaining-positive osteoblasts. B, Bone; BM, bone marrow. Scale bars, 1 mm (low magnification), 50 μm (medium magnification), and 50 μm (high magnification). **(B)** Representative images of HE staining and anti-human vimentin, anti-rat osteocalcin, anti-human osteocalcin immunostaining. Boundary lines of new bone are drawn on HE staining. Arrowheads indicate representative immunostaining-positive cells. XB, xenografted bone; NB, new bone; BM, bone marrow (stroma between xenografted bone). Scale bars, 500 μm (low magnification) and 200 μm (high magnification).

We investigated whether EP4 receptor is expressed in human bone tissue by immunostaining with EP4 antibody. The expression of EP4 was detected on osteoblasts of human vertebral bone. This result indicates that the implanted human bone tissue expressed the receptor that AKDS001 binds to (Figure 2A).

### 3.2 AKDS001 Promoted New Bone Formation by Human Osteoblasts in a Xenograft Model

**FIGURE 3:**
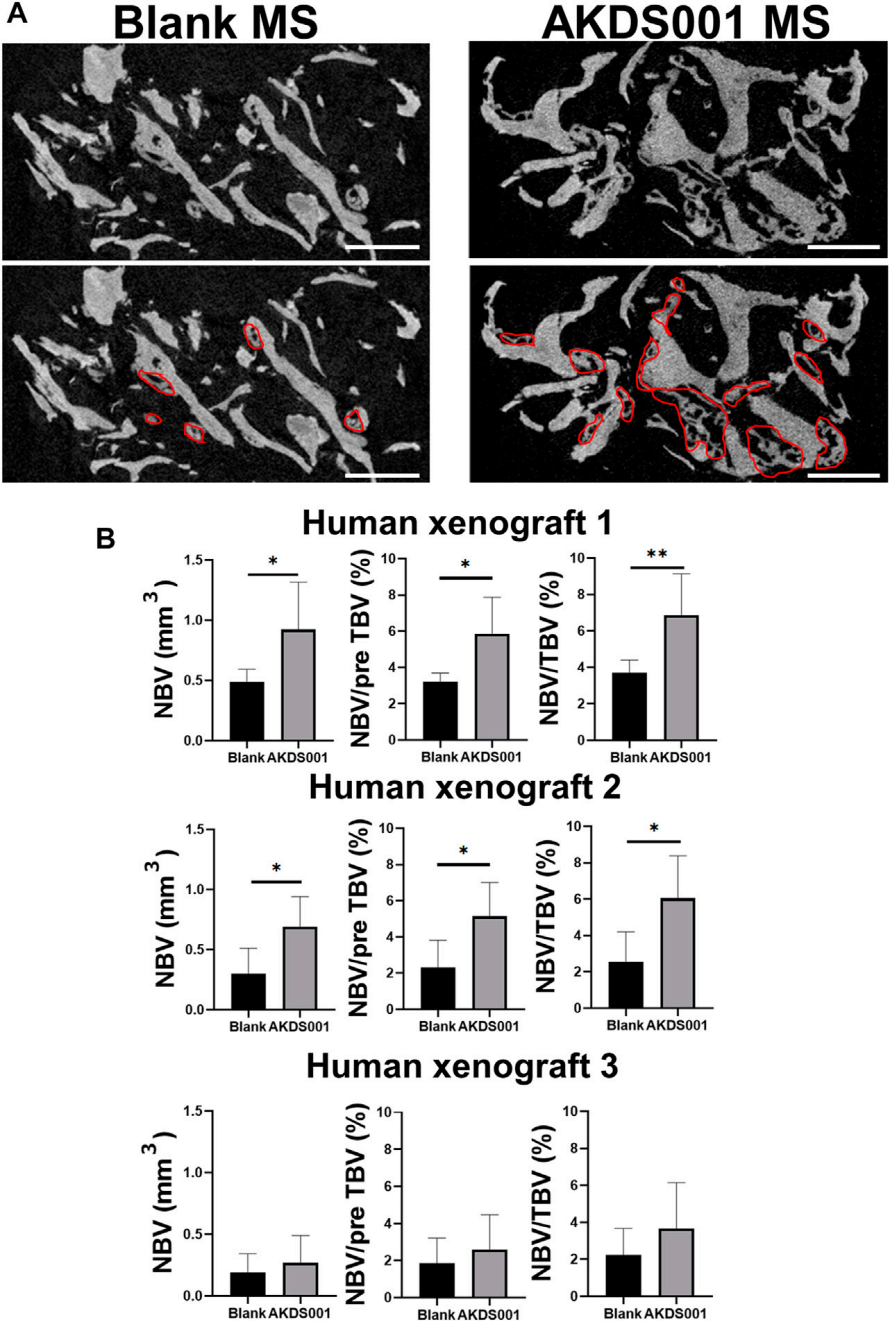
Microcomputed tomography (μCT) analysis of extracted bone. **(A)** Representative μCT images. Scale bars, 1 mm. The areas circled with red lines indicate new bone. **(B)** New bone volume (NBV), NBV/preoperative total bone volume (pre TBV) and NBV/total bone volume (TBV) were calculated for human xenograft 1–3. The 12 xenograft samples prepared from one patient were implanted. Blank MS or AKDS001 MS was mixed with the cultured bone (Blank MS, *n* = 6; AKDS001 MS, *n* = 6). Data represent the mean ± SD (error bars). Data were analyzed by two-tailed Student’s *t*-test (unpaired). *p < 0.05, **p < 0.01.

Next, we evaluated the effects of AKDS001 on *in vivo* bone regeneration using a xenograft model. The histological evaluation of the explanted sample at 4 weeks post-operation showed woven bone formation on the grafted bone (Figure 2B). A similar amount of vascularization was observed at the center of the xenografts in both the Blank MS group and the AKDS001 MS group (Supplementary Figure S3). The immunostaining with an anti-human-specific vimentin antibody demonstrated the survival of human cells around the xenografted bone in both the Blank MS group and the AKDS001 MS group (Supplementary Figure S2B). These cells were distributed on the surface of the newly formed woven bone. Next, to clarify the origin of the osteoblasts, immunostaining with antibodies specifically binding to human or rat osteocalcin was performed. The cross-species specificity of each antibody was confirmed using rat femur (Supplementary Figure S2). In both treatment groups, osteoblasts expressing human osteocalcin were predominant. These results suggest that the woven bone was predominantly formed by human osteoblasts. Extraction of the woven bone area based on the HU value of μCT data (Supplementary Figure S1E) showed that new bone formation increased in the AKDS001 MS group compared to the Blank MS group (Figures 3A,B). These results suggest that AKDS001 MS enhances new bone formation by human osteoblasts originating from the grafted bone.

### 3.3 AKDS001 MS Showed Bone Formation by Minimodeling

**FIGURE 4:**
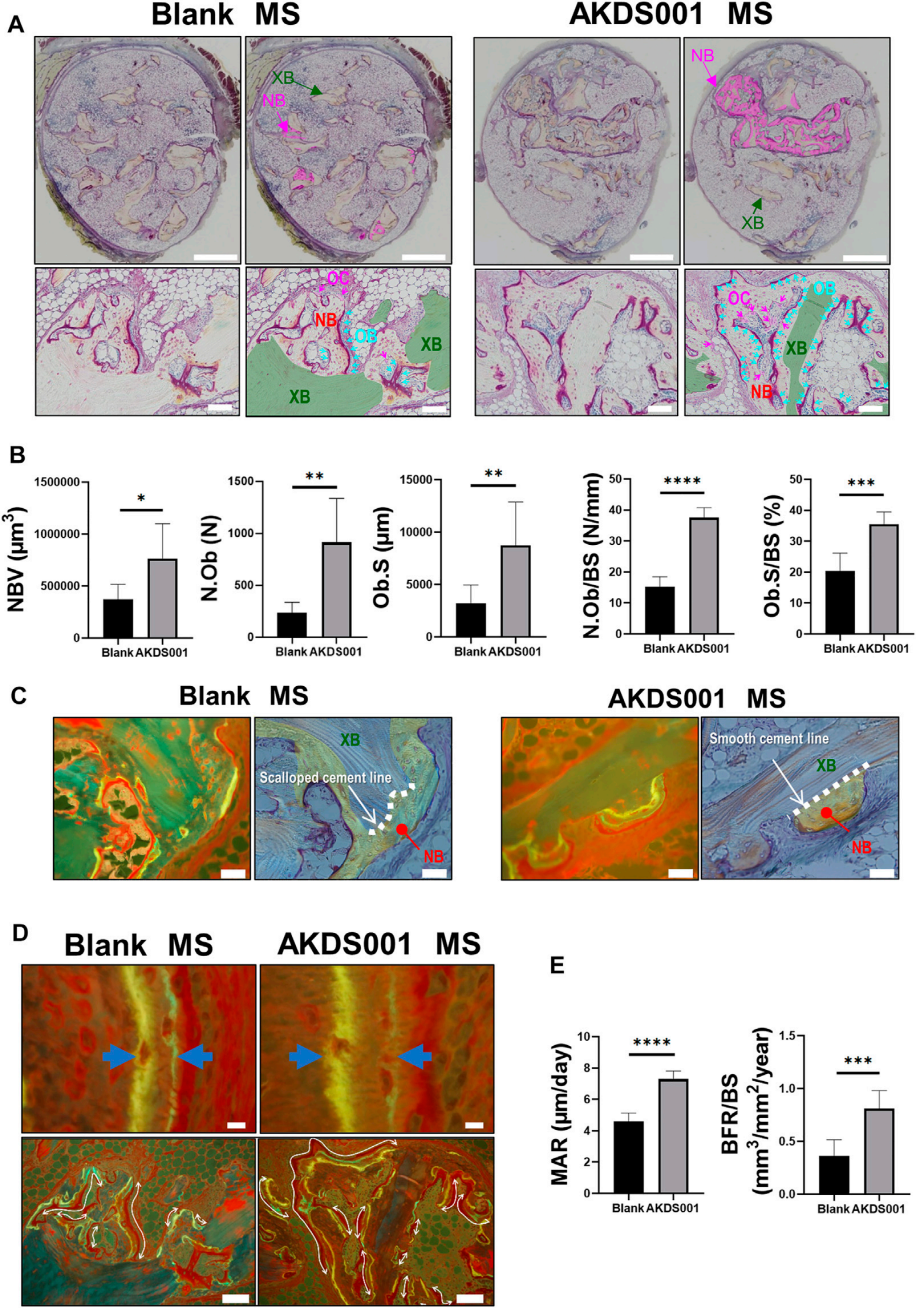
Histomorphometric analysis of Blank MS versus AKDS001 MS (1.0 mg/ml) in newly formed bone. **(A)** Representative images of Villanueva bone staining of extracted samples. Scale bars, 1 mm (upper) and 100 μm (lower). NB, new bone; XB, xenografted bone; OB, osteoblast; OC osteoclast. In the upper image, the NB area is painted with pink. In the lower image, the XB area is painted with green; Ob is colored blue (blue arrow); OC is colored pink (pink arrow). **(B)** New bone volume (NBV), number of osteoblasts (N.Ob), osteoblast surface (Ob.S), N.Ob/bone surface (BS), and Ob.S/BS on new bone. **(C)** Representative images of new bone formation. Villanueva bone staining (upper) and fluorescent image of the same area (lower). Scale bars, 50 μm. In the upper image, non-NB areas are filled with blue. The boundary between the grafted bone and the new bone is traced by the dotted line. **(D)** Representative fluorescent images of new bone. Scale bars, 10 μm (upper) and 100 μm (lower). The width of the double staining area is indicated by arrows (upper) and the double staining area is indicated by curves (lower). **(E)** Mineral apposition rate (MAR) and bone formation rate (BFR)/BS on new bone. *p < 0.05, **p < 0.01, ***p < 0.001, ****p < 0.0001 by two-tailed Student’s *t*-test (unpaired).

To clarify how AKDS001 MS increases new bone formation in the xenograft model, histomorphometric analysis on the area of newly formed bone was performed. Osteoblasts were more numerous and widely distributed in the AKDS001 MS group (Figures 4A,B). In the Blank MS group, most of the new bone formation was observed on scalloping cement lines created by osteoclastic bone resorption. In contrast, in the AKDS001 MS group, new bone was formed on smooth cement lines (Figure 4C). New bone formation on smooth cement lines is characteristic of bone minimodeling, in contrast to bone remodeling in which bone resorption by osteoclasts precedes bone formation by osteoblasts (Jee et al., 2007). Thus, the mode of osteogenesis caused by AKDS001 MS was shown to be minimodeling rather than remodeling.

### 3.4 AKDS001 MS Promoted Bone Formation by Increasing Mature Osteoblasts on New Bone

Bone formation parameters (MAR and BFR/BS) of the AKDS001 MS group were significantly increased on the surface of new bone compared with the Blank MS group (Figures 4D,E). Taken together, these results indicate that AKDS001 MS increased the number of mature osteoblasts and enhanced new bone formation.

### 3.5 AKDS001 MS Suppressed Resorption of Grafted Bone by Reducing Mature Osteoclasts

**FIGURE 5:**
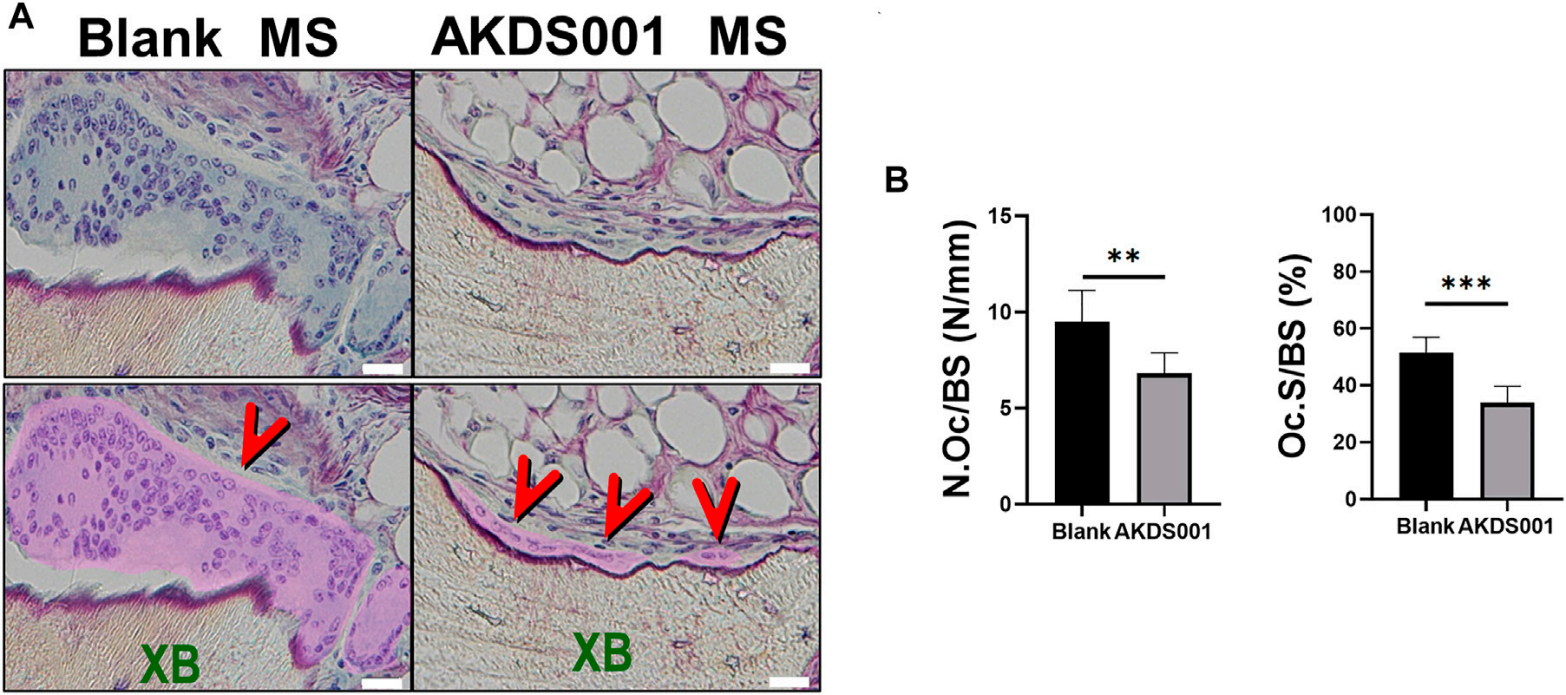
Histomorphometric analysis of Blank MS versus AKDS001 MS (1.0 mg/ml) on the surface of xenografted bone. **(A)** Representative Villanueva bone staining of xenografted bone. XB, xenografted bone. Scale bars, 20 μm. Osteoclast area is filled with pink and pointed by red arrows. **(B)** Number of osteoclasts (N.Oc)/BS and osteoclast surface (Oc.S)/BS on xenografted bone. Blank MS, *n* = 6; AKDS001 MS, *n* = 6. Data represent the mean ± SD (error bars). **p < 0.01, ***p < 0.001 by two-tailed Student’s *t*-test (unpaired).

To confirm the effect of AKDS001 MS on the resorption of xenografted bone, we performed histomorphometric analysis on the surface of xenografted bone, but not on the surface of new bone. Interestingly, the administration of AKDS001 MS reduced the absorption area, erosion area, and osteoclast number on the surface of xenografted bone (Figures 5A,B). These results suggest that AKDS001 MS suppresses the resorption of xenografted bone.

### 3.6 AKDS001 MS Promoted Bone Formation by Minimodeling in a Concentration-Dependent Manner

**FIGURE 6:**
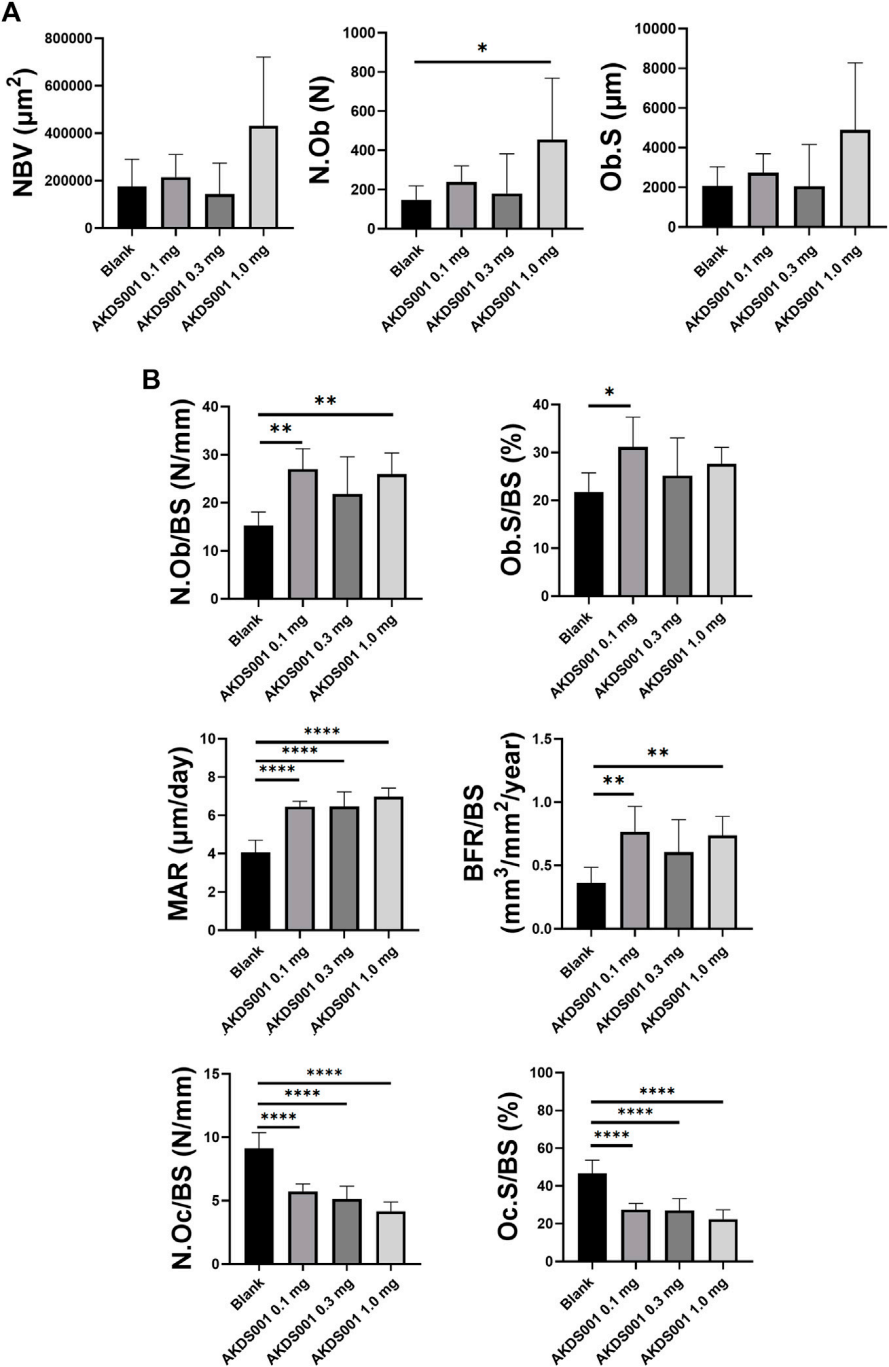
Histomorphometric analysis of Blank MS versus AKDS001 MS (0.1, 0.3, 1.0 mg/ml). **(A)** New bone volume (NBV), number of osteoblasts (N.Ob) and osteoblast surface (Ob.S). **(B)** N.Ob/bone surface (BS), Ob.S/BS, mineral apposition rate (MAR) and bone formation rate (BFR)/BS on new bone. Number of osteoclasts (N.Oc)/BS and osteoclast surface (Oc.S)/BS on xenografted bone. Blank MS, *n* = 6; AKDS001 MS 0.1 mg/ml, *n* = 6; AKDS001 MS 0.3 mg/ml, *n* = 6; AKDS001 MS 1.0 mg/ml, *n* = 6. *p < 0.05, **p < 0.01, ***p < 0.001, ****p < 0.0001 by Dunnett’s test.

Next, the concentration response by AKDS001 MS (0.1, 0.3, and 1.0 mg/ml) was evaluated by histomorphometric analysis using the xenograft model. At the highest concentration of AKDS001 MS (1.0 mg/ml), N.Ob significantly increased and NBV, Ob.S showed an increasing trend (Figure 6A), similar to the result of the preceding experiment (Blank MS vs. AKDS001 MS 1.0 mg/ml) (Figure 4B). N.Ob/BS, Ob.S/BS, MAR, and BFR/BS significantly increased even in the lowest concentration of AKDS001 MS (0.1 mg/ml) (Figure 6B). Also, N.Ob/BS, Ob.S/BS, MAR, and BFR/BS showed an increasing trend in all concentrations of AKDS001 MS (0.1, 0.3, and 1.0 mg/ml) (Figure 6B). Irrespective of the concentration, AKDS001 MS (0.1, 0.3, or 1.0 mg/ml) induced bone formation with a high density of osteoblasts and a high bone formation rate. As for the bone resorption parameters on the area without new bone formation, N.Oc/BS and Oc.S/BS decreased in a concentration-dependent manner (Figure 6B). Taken together, AKDS001 MS changed the characteristics of bone formation from remodeling to minimodeling, irrespective of the concentration, and AKDS001 MS led to the increase of new bone volume in a concentration-dependent manner.

### 3.7 AKDS001 Bound to Human EP4 With High Selectivity and Affinity

Next, we investigated the effect of AKDS001 on human cells in *in vitro* experiments. The human EP4 selectivity of AKDS001 was evaluated. AKDS001 showed Ki values of 6.0 × 10^–11^ mol/L for the EP4 receptor. EP4 selectivity values to EP1, EP2, and EP3 were 8,500-fold (Ki = 5.1 × 10^–7^ mol/L), 580-fold (Ki = 3.5 × 10^–8^ mol/L), and >167,000-fold (Ki = >1.0 × 10^–5^ mol/L), respectively. The efficacy of AKDS001 (EC_50_) for EP4-stimulated increase in intracellular cAMP concentrations was 4.2 × 10^–10^ mol/L, which was comparable to PGE2 (EC50 = 1.4 × 10^–9^ mol/L) and considerably higher than 5.7 × 10^–6^ mol/L and 1.3 × 10^–6^ mol/L of EC_50_ for EP2 and prostacyclin IP receptors, respectively (>3,000-fold). Furthermore, high specificity of AKDS001, up to 1 μmol/L, was demonstrated in safety screening for 87 off-targets (Supplementary Figures S4, S5). These results support the notion that AKDS001 is a highly potent and selective EP4 receptor agonist.

### 3.8 Functional Activity of AKDS001 on Human EP4 was Comparable to Its Activity in Rats

The effectiveness of AKDS001 was also investigated using CHO-K1 cells expressing EP4 in humans and rats. The EC_50_ of human and rat EP4 for PGE2 was 0.95 and 1.04 nM, respectively. The EC_50_ of that for AKDS001 was 0.92 and 1.27 nM, respectively. These results suggest that AKDS001 exerts similar functional activity between humans and rats.

### 3.9 AKDS001 Promoted Osteoblastic Differentiation of hMSC Through the cAMP/PKA Pathway

**FIGURE 7:**
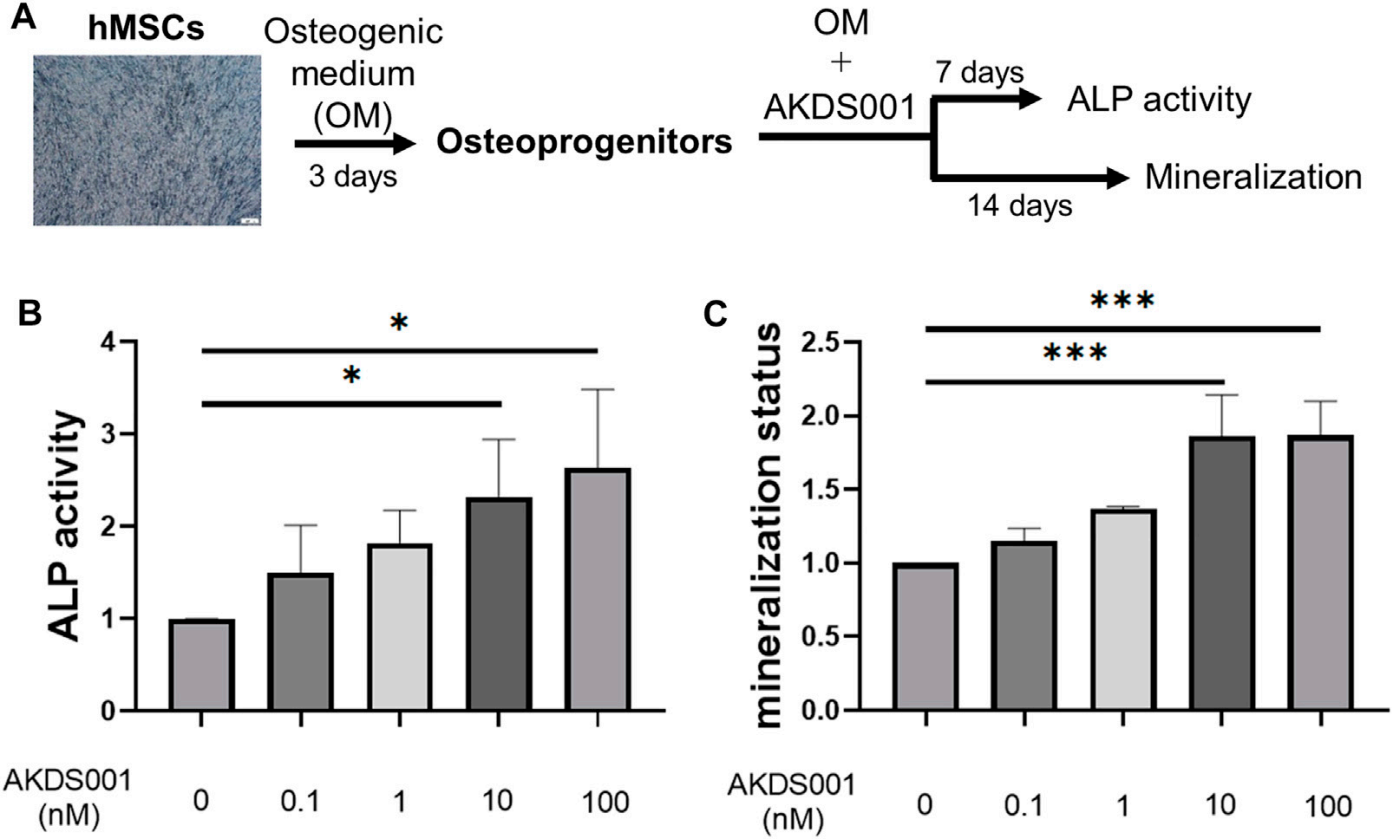
Effect of AKDS001 on human mesenchymal stem cells (hMSCs). **(A)** Scheme showing the protocol of osteoblastic differentiation. **(B)** ALP activity of hMSCs treated with osteogenic medium (OM) and AKDS001 (0–100 nM). ALP activity in the treatment group was expressed relative to that of the control group (AKDS001 0 nM). *n* = 3 per group. **(C)** Mineral apposition of hMSCs with OM and AKDS001 (0–100 nM). Mineral apposition in the treatment group was expressed relative to that of the control group (AKDS001 0 nM). *n* = 3 per group. *p < 0.05, ***p < 0.001 by Dunnett’s test.

To evaluate the effect of AKDS001 on osteoblastic differentiation, ALP activity and matrix mineralization were measured (Figure 7A). AKDS001 increased ALP activity and mineralization in a concentration-dependent manner from 1 to 100 nM (Figures 7B,C). These results showed that AKDS001 enhances the differentiation from hMSCs to osteoblasts. The major signal pathways downstream of EP4 are the cAMP/PKA pathway and the PI3K/β-catenin pathway (Yokoyama et al., 2013). To identify the responsible signal pathway for the enhancement of osteoblastic differentiation by AKDS001, an inhibition assay was performed using H89, a PKA inhibitor, and wortmannin, a PI3K inhibitor. The enhanced ALP activity by AKDS001 was suppressed by H89 in a concentration-dependent manner, but not by wortmannin (Supplementary Figures S6A, S3B). These results suggest that AKDS001 increases ALP activity through PKA. As PKA is activated by cAMP, the effect of cAMP on ALP activity was evaluated using dibutyryl (db) cAMP. ALP activity of hMSCs was increased through db cAMP (Supplementary Figure S6C). cAMP assay of hMSC revealed that AKDS001 increases cAMP production. The EC_50_ of cAMP production of AKDS001 was 28 nM. These results indicate that AKDS001 facilitates osteoblastic differentiation through the cAMP/PKA pathway.

### 3.10 AKDS001 Suppressed Differentiation of Human Osteoclast Precursors Into Mature Osteoclasts

**FIGURE 8:**
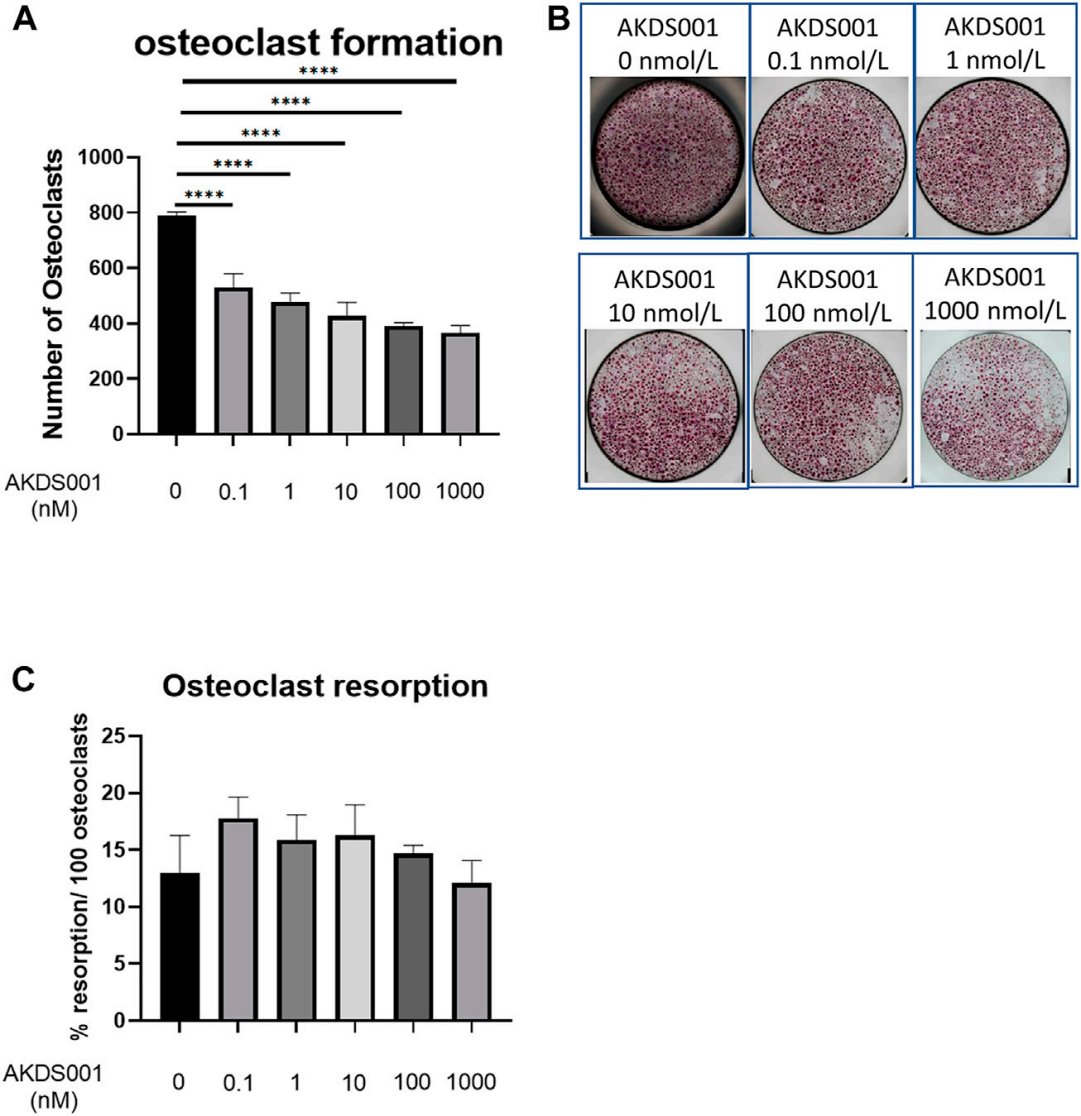
Effect of AKDS001 on human osteoclast precursors. **(A)** Number of osteoclasts after the induction of osteoclastic differentiation with or without AKDS001 (0.1–1000 nM). *n* = 4 per group. **(B)** Representative images of osteoclasts after the induction of osteoclastic differentiation with or without AKDS001 (0.1–1000 nM). **(C)** Resorption area by 100 osteoclasts induced by osteoclastic differentiation with or without AKDS001 (0.1–1000 nM). *n* = 4 per group. ****p < 0.0001 by Dunnett’s test.

To investigate the effect of AKDS001 on osteoclast lineage, an osteoclastic differentiation assay and an osteoclast resorption assay were performed. Multinucleated osteoclast numbers decreased depending on the concentrations of AKDS001 (Figures 8A,B). In contrast, no effect on the bone resorption capacity by osteoclasts was observed (Figure 8C). These results suggest that AKDS001 suppresses bone resorption by regulating the differentiation of osteoclast precursors into mature osteoclasts.

## 4 Discussion

**FIGURE 9:**
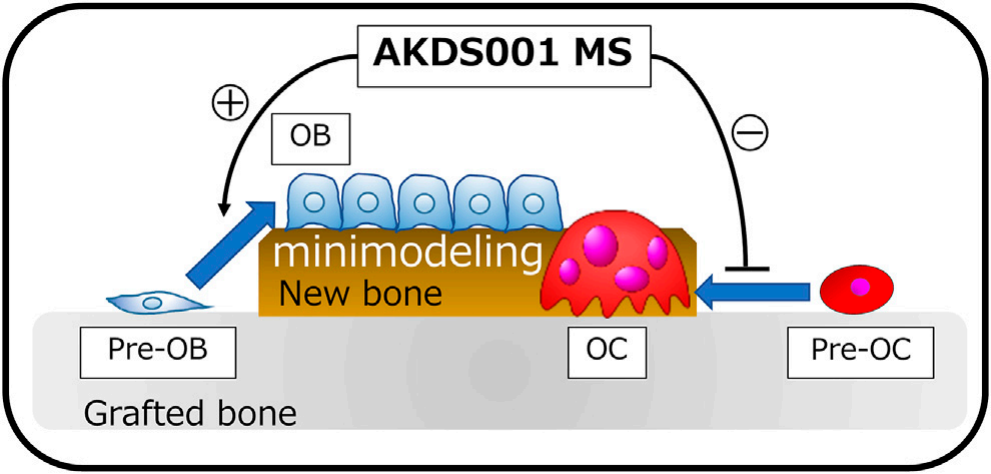
Schemas showing the osteogenic mechanisms induced by AKDS001 MS. AKDS001 promotes osteoblast differentiation and inhibits osteoclast maturation, resulting in new bone formation by minimodeling. MS, microsphere; MSC, mesenchymal stem cell; OB, osteoblast; OC, osteoclast.

EP4 receptors have been identified as promising targets for osteoporosis treatment and bone regeneration in both *in vitro* (Suzawa et al., 2000; Nakagawa et al., 2007; Downey et al., 2009; Minamizaki et al., 2009; Kanayama et al., 2018) and *in vivo* (Tanaka et al., 2004; Toyoda et al., 2005; Iwaniec et al., 2007; Downey et al., 2009; Kanayama et al., 2018) preclinical studies using a variety of animal species. However, the clinical therapeutic effect of EP4 receptor agonists on human bone tissue has not yet been proven. To address the clinical therapeutic effects of an EP4 agonist, we established a novel rat xenograft model using patient-derived bone tissue harvested during lumbar decompression surgery for degenerative spinal disease. In this study, we demonstrated for the first time that local sustained release of an EP4 agonist from MS mixed with xenograft bone tissue significantly stimulated the osteogenic response of human bone graft in a concentration-dependent manner. The process of bone formation by AKDS001 MS was shown to be minimodeling, in which bone formation occurs without preceding bone resorption rather than remodeling (Figure 9). The results of the xenograft model of human bone suggest that AKDS001 MS has the potential to be a novel osteogenic enhancer in combination with autogenous bone grafting.

The human bone xenograft model used in this study has the advantage of yielding homogeneous and stable human bone formation. The establishment of a stable human bone xenograft model is important because such a model could produce useful data in preclinical evaluations of medicines (Chong et al., 2016). Several previous reports have evaluated human cell–derived bone formation through the combined use of cultured cells such as MSCs and artificial scaffolds (Yoshikawa et al., 1996; Yoshikawa and Myoui, 2005; Wang et al., 2011; Liu et al., 2018). Bone formation by cultured cells is homogeneous and stable, but different from human bone formation *in vivo* because there is no bone tissue to act as a scaffold. To reproduce human bone formation *in vivo*, xenografting of the human bone itself is an important step to predict the effects in humans. Surowiec et al. xenografted patient bone typically discarded as surgical waste into athymic mice in order to evaluate the effect of sclerostin antibodies in pediatric osteogenesis imperfecta patients (Surowiec et al., 2020). They roughly trimmed the xenografted bone immediately after excision, and confirmed the survival of human cells until 12 weeks after xenografting (Surowiec et al., 2020). The results were heterogeneous from sample to sample and the amount of newly formed bone was very small, however (Surowiec et al., 2020). To ensure the uniformity of grafting samples and enhance human bone formation by increasing the surface area of the grafting bone, we established a novel human xenograft model by pulverizing trimmed cancellous bone into small pieces and pre-culturing human bone before implanting and confirmed its reproducibility.

In the xenograft bone model, AKDS001 MS reduced the absorption of grafted bone and promoted new bone formation by minimodeling. Normally, new bone is formed by remodeling, in which bone resorption by osteoclasts precedes bone formation by osteoblasts (Langdahl et al., 2016). AKDS001 MS changed the mode of bone formation from remodeling to minimodeling, by which new bone is formed without preceding bone resorption (Figure 9) (Langdahl et al., 2016). AKDS001 MS promoted the recruitment of mature osteoblasts, enhanced the bone formation rate, and suppressed the recruitment of mature osteoclasts. The effect of AKDS001 MS to increase osteoblasts and bone formation is similar to the effects of the other EP4 agonists (Ito et al., 2006; Ke et al., 2006; Iwaniec et al., 2007). However, unlike AKDS001, some EP4 agonists are reported to increase osteoclast recruitment (Ito et al., 2006; Iwaniec et al., 2007). Taken together, when AKDS001 is combined with autogenous bone grafting, AKDS001 MS is expected to preserve autogenous bone as a scaffold for bone formation by suppressing bone resorption and is expected to increase the osteogenic activity of autogenous bone by enhancing osteoblast activity. Autogenous bone grafting is widely used in surgery for large bone defects and spinal fusion (Bauer and Muschler, 2000; Dimitriou et al., 2011; Makino et al., 2018). The number of patients undergoing surgery, spinal fusion surgeries in particular, is increasing as a result of the ageing population. In these patients, the quality of autogenous bone deteriorates in terms of bone volume and osteogenic activity (Infante and Rodríguez, 2018). The combined use of AKDS001 MS and autogenous bone (biologically enhanced autograft) has the potential to enhance the osteogenic capacity in autogenous bone grafting in older adults.

This study has several limitations. First, the xenograft implantation in this study was performed at a subcutaneous lesion (ectopic model) instead of at bony sites (orthotopic model). We chose subcutaneous implantation because osteoblastic cell migration from surrounding bone tissue makes it difficult to show the effects of AKDS001 on the implanted human cells and tissues. Further study using preclinical models is needed. Second, xenografts were pre-cultured for 2 weeks before implantation. Without pre-culture, the results vary from sample to sample (Surowiec et al., 2020) and pre-culture increases the osteogenic activity of cells *in vivo* (Yoshikawa et al., 1996). Pre-culture was needed to stabilize the number of viable cells and to increase the bone formation in this study because autogenous bone is affected by storage conditions after sampling (Surowiec et al., 2020) and the subcutaneous environment is unfavorable for bone formation. To overcome this gap between the model and clinical practice, a rat model spinal fusion was conducted using allografts without pre-culture and this confirmed the enhancement of bone regeneration by ADKS001 MS (Okada et al., 2021). Also, we are currently investigating the effects of AKDS001 MS at load-bearing locations (spine and lower limbs). We hope that these experiments will further validate the therapeutic value of AKDS001 on bone regeneration.

## 5 Conclusion

We established a novel xenograft model of human bone and demonstrated the osteogenic effects of AKDS001 on human cells and human bone *in vivo* (Figure 9). The mode of new bone formation by AKDS001 MS was minimodeling in which the volume of grafted bone is preserved (Figure 9). The combined use of an EP4 agonist and autogenous bone (biologically enhanced autograft) may be a novel option for bone grafting surgery. However, we should be careful in interpreting the results because the results because male xenografts were implanted in male rats in the present study. It remains to be seen whether females can benefit from the positive effects of AKDS001 MS by using female xenografts implanted in female rats in clinically relevant animal models.

## Data Availability

The raw data supporting the conclusion of this article will be made available by the authors, without undue reservation.

## Supplementary Material

Supplementary Figure S1 Extraction of new bone formation area Representative images of xenografted bone in the AKDS001 MS group. **(A)** HE staining. **(B)** Original microcomputed tomography (CT) image. **(C)** Color map CT image based on Hounsfield unit values. **(D)** Extracted new bone formation area by analysis processing. Scale bars, 100 μm. **(E)** Color map CT image (upper) and new bone formation area (lower) of Figure 3A. Scale bars, 1 mm.

Supplementary Figure S2 Representative images of immunostaining using anti-human vimentin, anti-human osteocalcin, anti-rat osteocalcin for rat femur and human vertebral lamina. Scale bars, 200 μm.

Supplementary Figure S3 Vascularization in xenografts Hematoxylin and eosin (HE) staining at low magnification (upper) and high magnification (center), and CD31 immunostaining at high magnification (lower). Scale bars, 500 μm (low magnification) and 100 μm (high magnification).

Supplementary Figure S4 Off-targets of AKDS001 (1) Off-target effects on G protein-coupled receptor, transporters, ion channels, and nuclear receptors by AKDS001.

Supplementary Figure S5 Off-targets of AKDS001 (2) Off-target effects on kinases, other non-kinase enzymes by AKDS001.

Supplementary Figure S6 Evaluation of pathways involved in the osteogenic effects of AKDS001 **(A)** ALP activity of hMSCs treated with or without AKDS001 (100 nM) and PKA inhibitor, H89 (5, 10, 20 μM). *n* = 3 per group. **(B)** ALP activity of hMSCs treated with or without AKDS001 (100 nM) and PI3K inhibitor, wortmannin (20, 100, 500 nM). *n* = 3 per group. **(C)** ALP activity of hMSCs treated with or without dibutyryl-cAMP (db cAMP) (0.5, 1, 2 mM). *n* = 3 per group. **p* < 0.05, ***p* < 0.01 by Dunnett’s test. ns: not statistically significant.

## References

[B1] Bauer T. W. Muschler G. F. (2000). Bone Graft Materials. Clin. Orthopaedics Relat. Res. 371, 10–27. 10.1097/00003086-200002000-00003 10693546

[B2] Chong M. S. K. Bao C. Ng K. P. Lim J. Chan J. K. Y. (2016). Human Bone Xenografts: from Preclinical Testing for Regenerative Medicine to Modeling of Diseases. Curr. Mol. Bio Rep. 2 (3), 158–170. 10.1007/s40610-016-0044-4

[B3] Dillon J. P. Waring-Green V. J. Taylor A. M. Wilson P. J. M. Birch M. Gartland A. (2012). Primary Human Osteoblast Cultures. Methods Mol. Biol. 816, 3–18. 10.1007/978-1-61779-415-5_1 22130918

[B4] Dimitriou R. Jones E. McGonagle D. Giannoudis P. V. (2011). Bone Regeneration: Current Concepts and Future Directions. BMC. Med. 9, 66. 10.1186/1741-7015-9-66 21627784 PMC3123714

[B5] Downey M. E. Holliday L. S. Aguirre J. I. Wronski T. J. (2009). *In Vitro* and *In Vivo* Evidence for Stimulation of Bone Resorption by an EP4 Receptor Agonist and Basic Fibroblast Growth Factor: Implications for Their Efficacy as Bone Anabolic Agents. Bone 44 (2), 266–274. 10.1016/j.bone.2008.10.041 19013265 PMC2663525

[B6] García-Gareta E. Coathup M. J. Blunn G. W. (2015). Osteoinduction of Bone Grafting Materials for Bone Repair and Regeneration. Bone 81, 112–121. 10.1016/j.bone.2015.07.007 26163110

[B7] Hua Y. Su Y. Zhang H. Liu N. Wang Z. Gao X. (2021). Poly(lactic-co-glycolic Acid) Microsphere Production Based on Quality by Design: a Review. Drug Deliv. 28 (1), 1342–1355. 10.1080/10717544.2021.1943056 34180769 PMC8245074

[B8] Infante A. Rodríguez C. I. (2018). Osteogenesis and Aging: Lessons from Mesenchymal Stem Cells. Stem Cel Res. Ther. 9 (1), 244. 10.1186/s13287-018-0995-x PMC6158877 30257716

[B9] Ito M. Nakayama K. Konaka A. Sakata K. Ikeda K. Maruyama T. (2006). Effects of a Prostaglandin EP4 Agonist, ONO-4819, and Risedronate on Trabecular Microstructure and Bone Strength in Mature Ovariectomized Rats. Bone 39 (3), 453–459. 10.1016/j.bone.2006.02.054 16581323

[B10] Iwaniec U. T. Moore K. Rivera M. F. Myers S. E. Vanegas S. M. Wronski T. J. (2007). A Comparative Study of the Bone-Restorative Efficacy of Anabolic Agents in Aged Ovariectomized Rats. Osteoporos. Int. 18 (3), 351–362. 10.1007/s00198-006-0240-9 17120182

[B11] James A. W. LaChaud G. Shen J. Asatrian G. Nguyen V. Zhang X. (2016). A Review of the Clinical Side Effects of Bone Morphogenetic Protein-2. Tissue Eng. Part. B Rev. 22 (4), 284–297. 10.1089/ten.TEB.2015.0357 26857241 PMC4964756

[B12] Jee W. S. Tian X. Y. Setterberg R. B. (2007). Cancellous Bone Minimodeling-Based Formation: a Frost, Takahashi Legacy. J. Musculoskelet. Neuronal Interact. 7 (3), 232–239. 17947806

[B13] Kanayama S. Kaito T. Kitaguchi K. Ishiguro H. Hashimoto K. Chijimatsu R. (2018). ONO-1301 Enhances *In Vitro* Osteoblast Differentiation and *In Vivo* Bone Formation Induced by Bone Morphogenetic Protein. Spine 43 (11), E616–e624. 10.1097/brs.0000000000002439 29016438

[B14] Ke H. Z. Crawford D. T. Qi H. Simmons H. A. Owen T. A. Paralkar V. M. (2006). A Nonprostanoid EP4 Receptor Selective Prostaglandin E2 Agonist Restores Bone Mass and Strength in Aged, Ovariectomized Rats. J. Bone Miner. Res. 21 (4), 565–575. 10.1359/jbmr.051110 16598377

[B15] Kodama J. Harumningtyas A. A. Ito T. Michlíček M. Sugimoto S. Kita H. (2021). Amine Modification of Calcium Phosphate by Low-Pressure Plasma for Bone Regeneration. Sci. Rep. 11 (1), 17870. 10.1038/s41598-021-97460-8 34504247 PMC8429709

[B16] Langdahl B. Ferrari S. Dempster D. W. (2016). Bone Modeling and Remodeling: Potential as Therapeutic Targets for the Treatment of Osteoporosis. Ther. Adv. Musculoskelet. Dis. 8 (6), 225–235. 10.1177/1759720x16670154 28255336 PMC5322859

[B17] Li M. Thompson D. D. Paralkar V. M. (2007). Prostaglandin E(2) Receptors in Bone Formation. Int. Orthop. 31 (6), 767–772. 10.1007/s00264-007-0406-x 17593365 PMC2266676

[B18] Liu C. C. Hu S. Chen G. Georgiou J. Arns S. Kumar N. S. (2015). Novel EP4 Receptor Agonist-Bisphosphonate Conjugate Drug (C1) Promotes Bone Formation and Improves Vertebral Mechanical Properties in the Ovariectomized Rat Model of Postmenopausal Bone Loss. J. Bone Miner. Res. 30 (4), 670–680. 10.1002/jbmr.2382 25284325

[B19] Liu Y. Yao Q. Sun H. (2018). Prostaglandin E2 Modulates Bone Morphogenetic Protein-2 Induced Osteogenic Differentiation on a Biomimetic 3D Nanofibrous Scaffold. J. Biomed. Nanotechnol. 14 (4), 747–755. 10.1166/jbn.2018.2490 31352948

[B20] Makino T. Tsukazaki H. Ukon Y. Tateiwa D. Yoshikawa H. Kaito T. (2018). The Biological Enhancement of Spinal Fusion for Spinal Degenerative Disease. Int. J. Mol. Sci. 19 (8). 10.3390/ijms19082430 PMC6121547 30126106

[B21] Minamizaki T. Yoshiko Y. Kozai K. Aubin J. E. Maeda N. (2009). EP2 and EP4 Receptors Differentially Mediate MAPK Pathways Underlying Anabolic Actions of Prostaglandin E2 on Bone Formation in Rat Calvaria Cell Cultures. Bone 44 (6), 1177–1185. 10.1016/j.bone.2009.02.010 19233324

[B22] Nakagawa K. Imai Y. Ohta Y. Takaoka K. (2007). Prostaglandin E2 EP4 Agonist (ONO-4819) Accelerates BMP-Induced Osteoblastic Differentiation. Bone 41 (4), 543–548. 10.1016/j.bone.2007.06.013 17681894

[B23] Okada R. Yamamori N. Nishida M. Ukon Y. Kaito T. (2021). A Novel EP4 Agonist (AKDS001) Enhances Spinal Interbody Fusion without Affecting Local Side Effects. J. Bone Miner. Res. 36 (Suppl. 1). Available at https://www.asbmr.org/meetings/annualmeeting/AbstractDetail?aid=94065fca-72d6-4f0f-b2e6-78d886946bc7 (Accessed December 8, 2021).

[B24] Raisz L. G. Pilbeam C. C. Fall P. M. (1993). Prostaglandins: Mechanisms of Action and Regulation of Production in Bone. Osteoporos. Int. 3 (Suppl. 1), 136–140. 10.1007/bf01621888 8461541

[B25] Surowiec R. K. Battle L. F. Ward F. S. Schlecht S. H. Khoury B. M. Robbins C. (2020). A Xenograft Model to Evaluate the Bone Forming Effects of Sclerostin Antibody in Human Bone Derived from Pediatric Osteogenesis Imperfecta Patients. Bone 130, 115118. 10.1016/j.bone.2019.115118 31678490 PMC6918492

[B26] Suzawa T. Miyaura C. Inada M. Maruyama T. Sugimoto Y. Ushikubi F. (2000). The Role of Prostaglandin E Receptor Subtypes (EP1, EP2, EP3, and EP4) in Bone Resorption: an Analysis Using Specific Agonists for the Respective EPs. Endocrinology 141 (4), 1554–1559. 10.1210/endo.141.4.7405 10746663

[B27] Tanaka M. Sakai A. Uchida S. Tanaka S. Nagashima M. Katayama T. (2004). Prostaglandin E2 Receptor (EP4) Selective Agonist (ONO-4819.CD) Accelerates Bone Repair of Femoral Cortex after Drill-Hole Injury Associated with Local Upregulation of Bone Turnover in Mature Rats. Bone 34 (6), 940–948. 10.1016/j.bone.2004.01.002 15193540

[B28] Toyoda H. Terai H. Sasaoka R. Oda K. Takaoka K. (2005). Augmentation of Bone Morphogenetic Protein-Induced Bone Mass by Local Delivery of a Prostaglandin E EP4 Receptor Agonist. Bone 37 (4), 555–562. 10.1016/j.bone.2005.04.042 16027058

[B29] Ukon Y. Makino T. Kodama J. Tsukazaki H. Tateiwa D. Yoshikawa H. (2019). Molecular-based Treatment Strategies for Osteoporosis: a Literature Review. Int. J. Mol. Sci. 20 (10). 10.3390/ijms20102557 PMC6567245 31137666

[B30] Ukon Y. Nishida M. Yamamori N. Shikanai D. Shirai T. Okada R. (2021). Prostaglandin EP4 Selective Agonist AKDS001 Enhances New Bone Formation by Minimodeling in a Heterotopic Xenograft Model of Human Bone. J. Bone Miner. Res. 36 (Suppl. 1). Available at https://www.asbmr.org/meetings/annualmeeting/AbstractDetail?aid=c9c4931e-18ac-40b9-84ea-4b8346b44820 (Accessed December 8, 2021).

[B31] Wang X. J. Huang H. Yang F. Xia L. G. Zhang W. J. Jiang X. Q. (2011). Ectopic Study of Tissue-Engineered Bone Complex with Enamel Matrix Proteins, Bone Marrow Stromal Cells in Porous Calcium Phosphate Cement Scaffolds, in Nude Mice. Cell Prolif 44 (3), 274–282. 10.1111/j.1365-2184.2011.00750.x 21535268 PMC6496638

[B32] Wilson R. J. Rhodes S. A. Wood R. L. Shield V. J. Noel L. S. Gray D. W. (2004). Functional Pharmacology of Human Prostanoid EP2 and EP4 Receptors. Eur. J. Pharmacol. 501 (1), 49–58. 10.1016/j.ejphar.2004.08.025 15464062

[B33] Yokoyama U. Iwatsubo K. Umemura M. Fujita T. Ishikawa Y. (2013). The Prostanoid EP4 Receptor and its Signaling Pathway. Pharmacol. Rev. 65 (3), 1010–1052. 10.1124/pr.112.007195 23776144

[B34] Yoshikawa H. Myoui A. (2005). Bone Tissue Engineering with Porous Hydroxyapatite Ceramics. J. Artif. Organs 8 (3), 131–136. 10.1007/s10047-005-0292-1 16235028

[B35] Yoshikawa T. Ohgushi H. Tamai S. (1996). Immediate Bone Forming Capability of Prefabricated Osteogenic Hydroxyapatite. J. Biomed. Mater. Res. 32 (3), 481–492. 10.1002/(sici)1097-463610.1002/(sici)1097-4636(199611)32:3<481::aid-jbm23>3.0.co;2-i 8897155

