# Supplementary figures and images for: Prostaglandin EP4 Selective Agonist AKDS001 Enhances New Bone Formation by Minimodeling in a Rat Heterotopic Xenograft Model of Human Bone
Source: Front Bioeng Biotechnol. 2022 Mar 17;10:845716. doi: 10.3389/fbioe.2022.845716 (PMC8968459; doi:10.3389/fbioe.2022.845716)

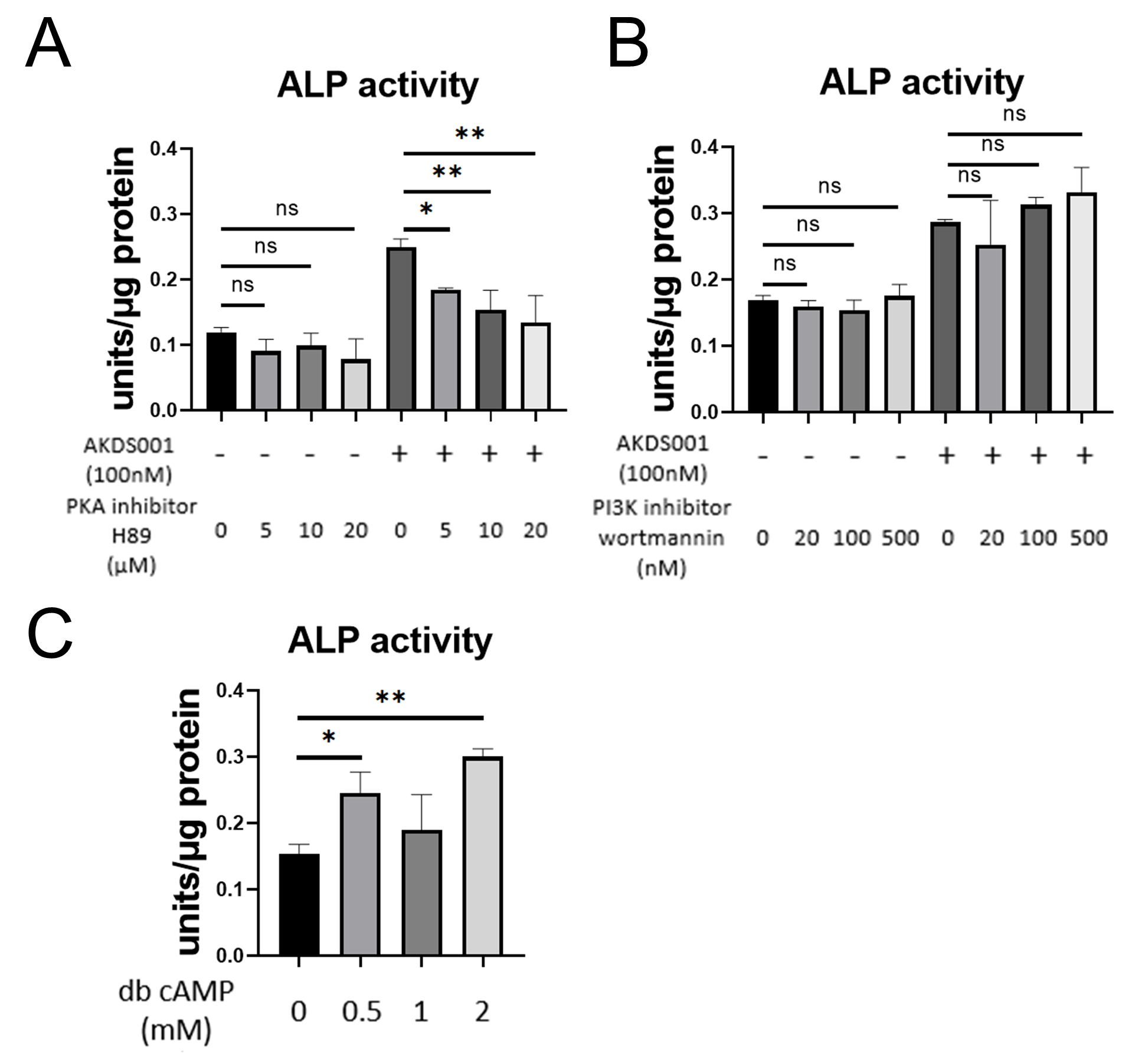

Supplement: Supplementary file 1 [file Image6.TIF]

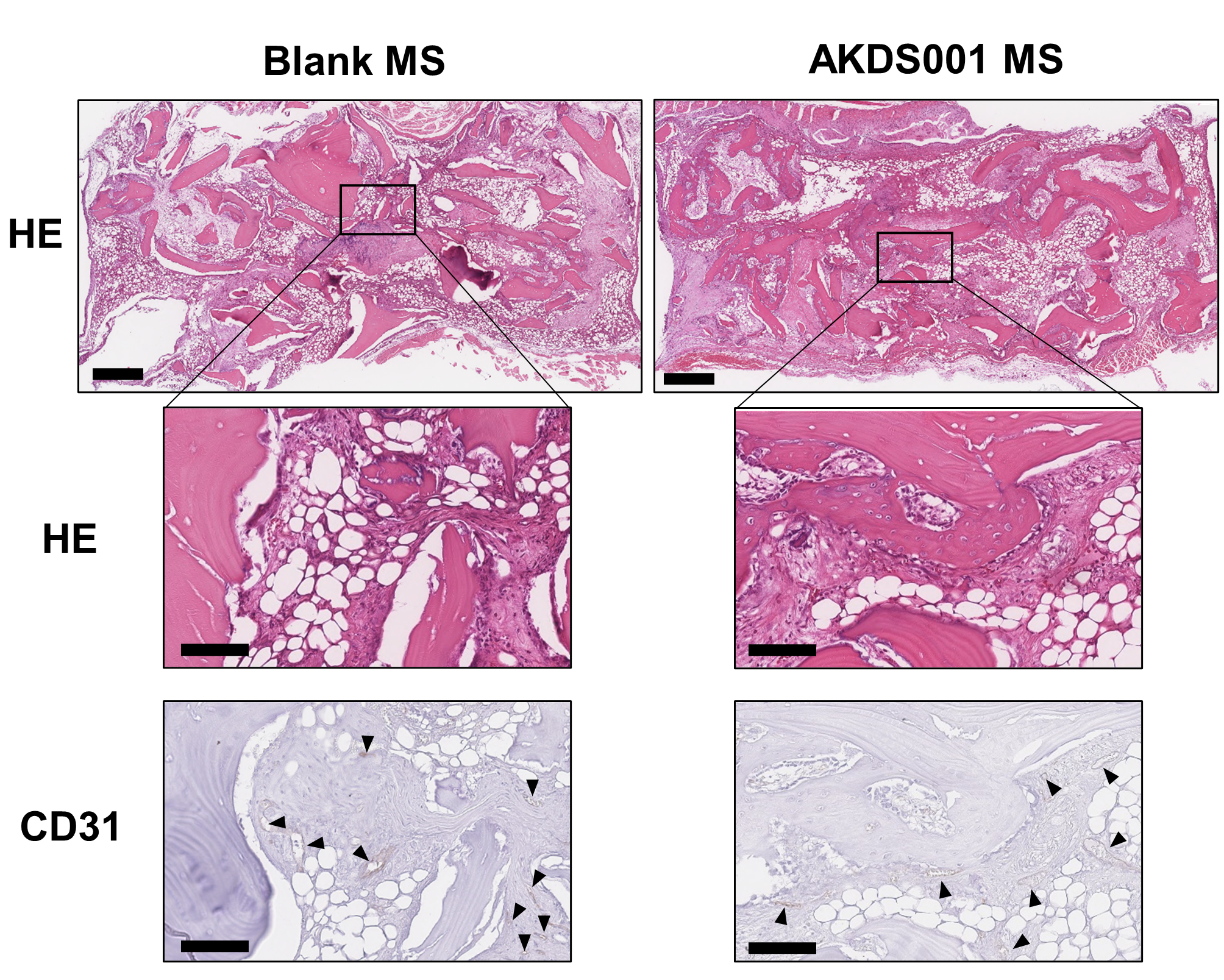

Supplement: Supplementary file 2 [file Image3.TIF]

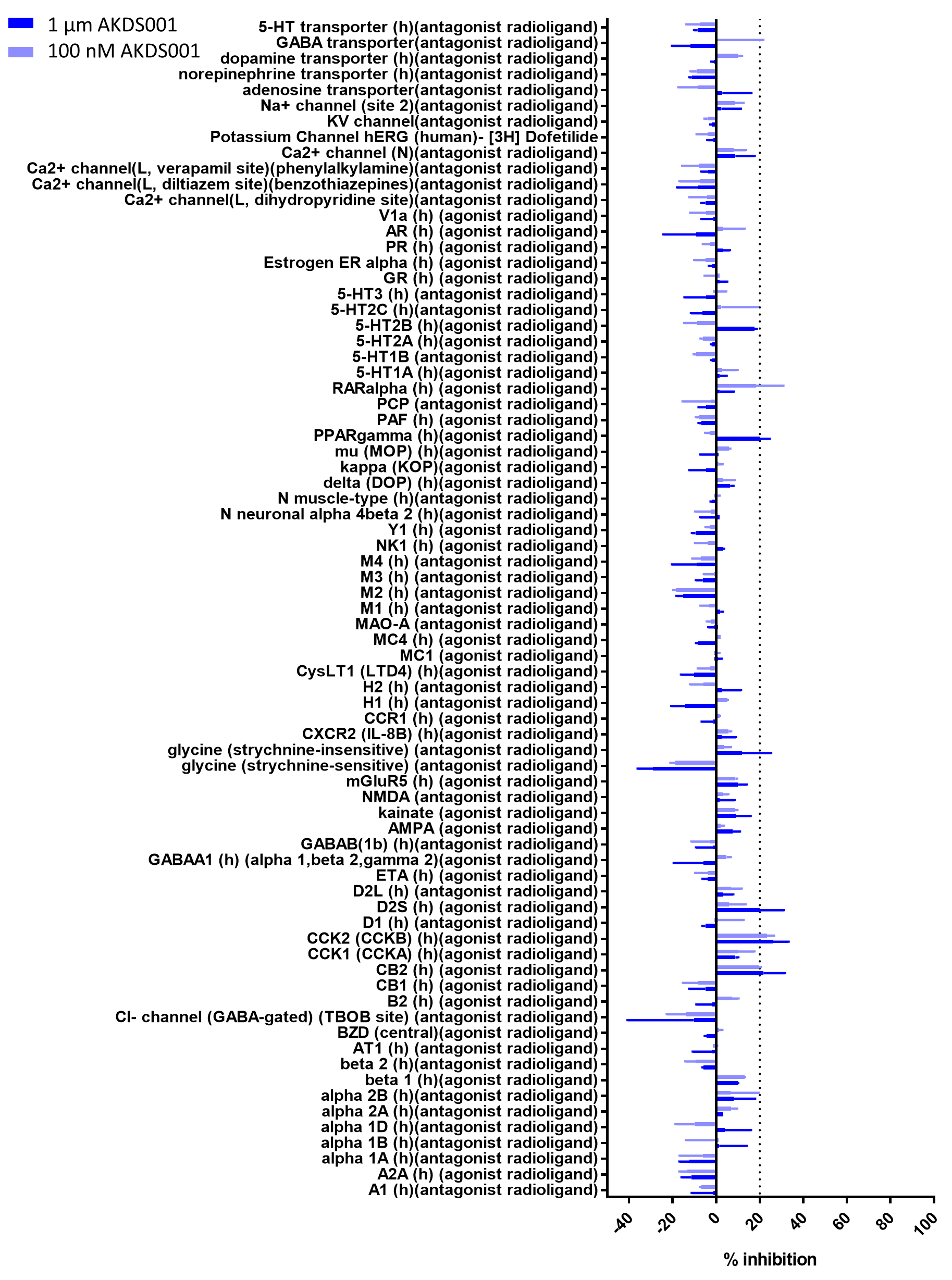

Supplement: Supplementary file 3 [file Image4.TIF]

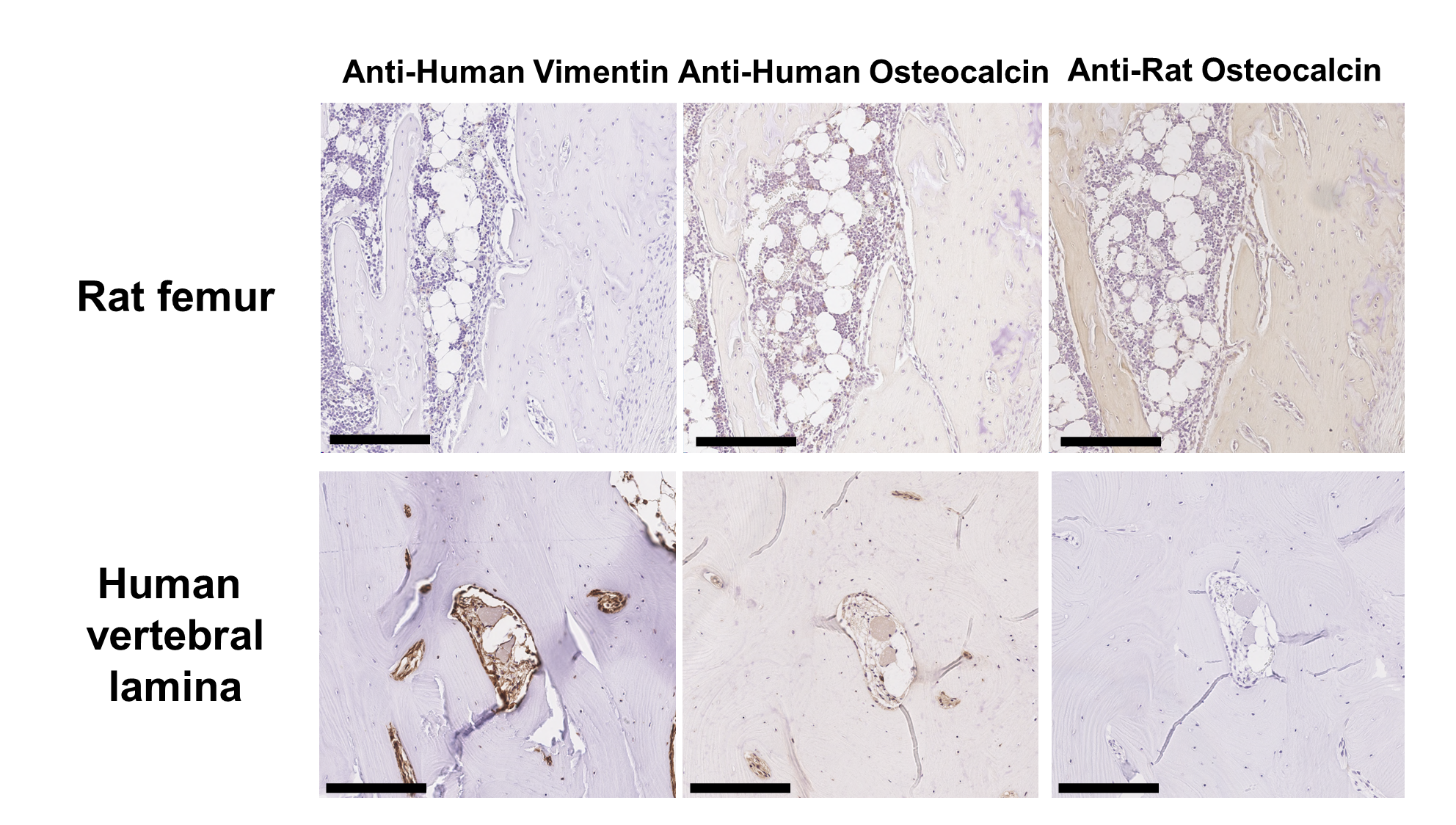

Supplement: Supplementary file 4 [file Image2.TIF]

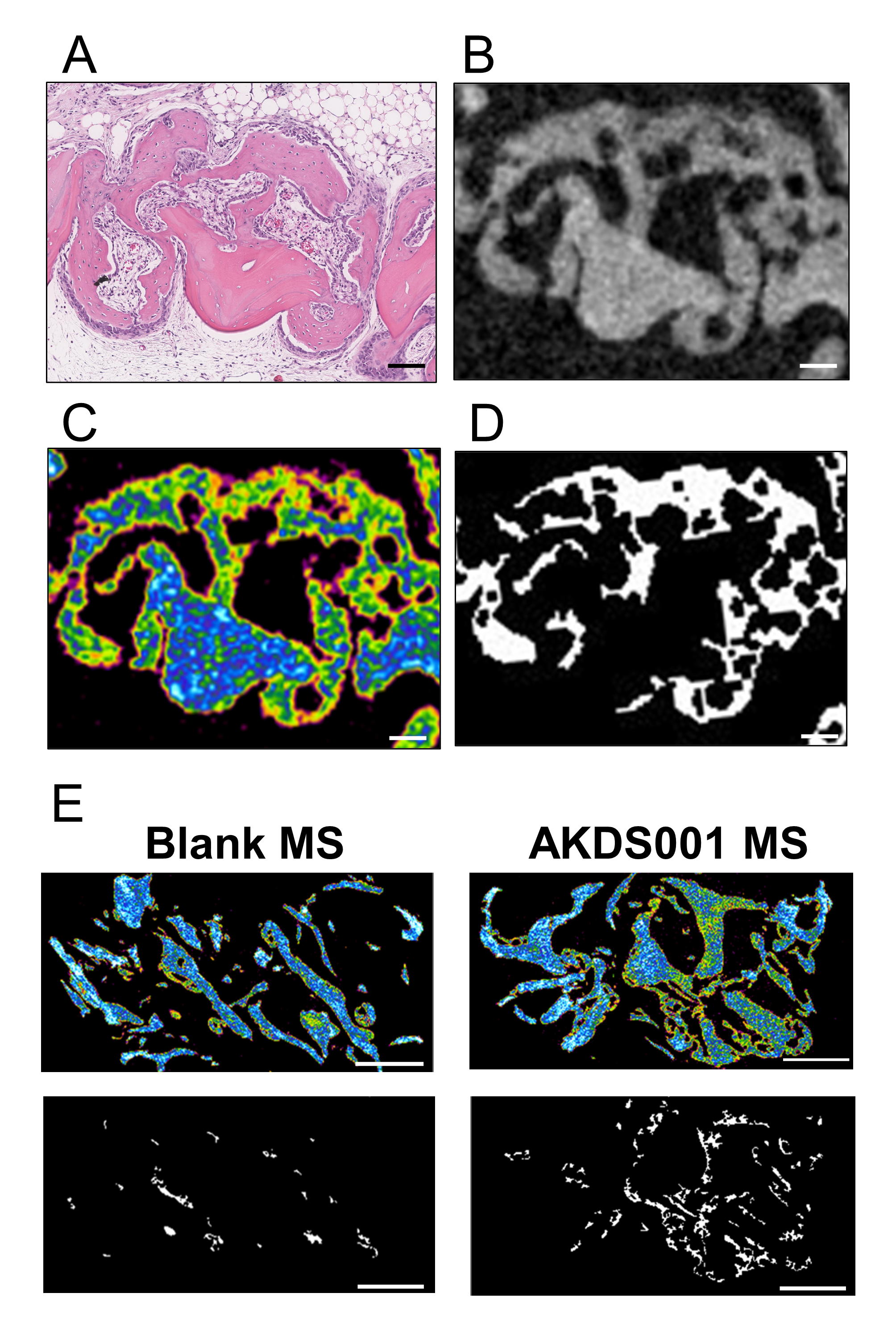

Supplement: Supplementary file 5 [file Image1.TIF]

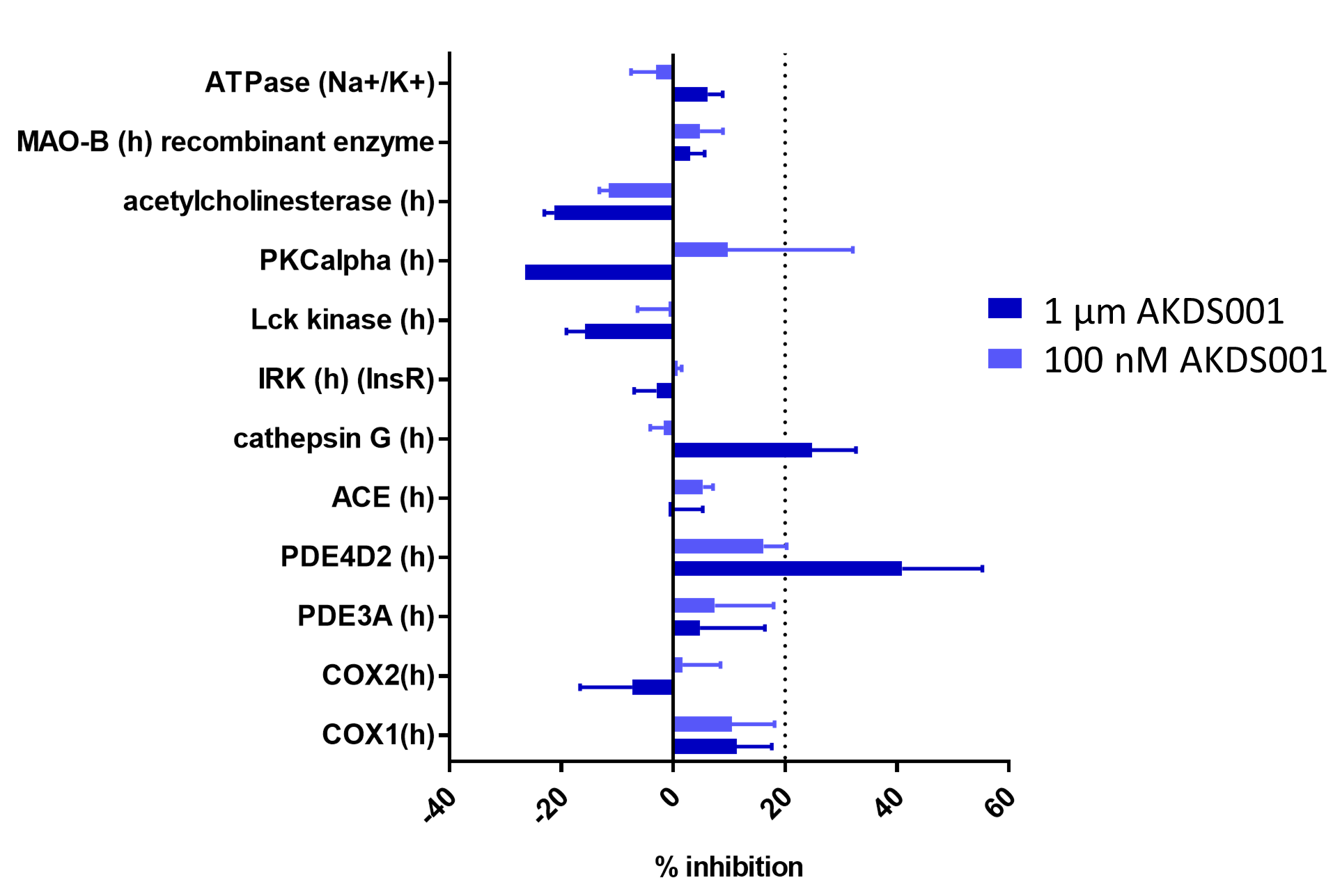

Supplement: Supplementary file 6 [file Image5.TIF]
